# Impact of Plasma Ashing on Mixed Monolayer Doping of Silicon

**DOI:** 10.1002/smtd.70970

**Published:** 2026-08-17

**Authors:** Pol Torres‐Vila, Thilo Glatzel, Mounir Mensi, Giovanni Boero, Juergen Brugger, Arnaud Bertsch

**Affiliations:** ^1^ Microsystems Laboratory École Polytechnique Fédérale de Lausanne (EPFL) Lausanne Switzerland; ^2^ Department of Physics University of Basel Basel Switzerland; ^3^ X‐Ray Diffraction and Surface Analytics Platform (XRD‐SAP), ISIC EPFL Valais Sion Switzerland

**Keywords:** dopant activation, KPFM, mixed monolayer doping, O_2_ plasma ashing, silicon doping, work function, XPS

## Abstract

Mixed monolayer doping (MMLD) is an attractive strategy for controllable low‐dose, low‐energy silicon doping via surface chemistry, yet the influence of carbon removal and surface effects on electronic properties remains unclear. In this work, silicon is doped using mixed monolayers of allyldiphenylphosphine (ADP) and 1‐undecene, followed by O_2_ plasma ashing, SiO_2_ capping, and rapid thermal annealing (RTA). The plasma treatment is systematically investigated, identifying conditions that effectively remove carbon while largely preserving phosphorus in the grafted layer, as confirmed by x‐ray photoelectron spectroscopy (XPS). Kelvin probe force microscopy (KPFM) reveals a pronounced decrease in work function (WF) with increasing ADP molar fraction, and the SiO_2_ deposition method plays a key role: evaporated SiO_2_ combined with O_2_ plasma leads to a WF consistent with enhanced n‐type activation, whereas sputtered SiO_2_ induces strong work‐function pinning, independent of ADP concentration. Four‐point probe and Hall measurements confirm increasing conductivity and sheet carrier density with ADP fraction, but show no significant dependence on plasma treatment, indicating that carbon‐related effects are confined to the near‐surface region. These results demonstrate that surface‐sensitive WF changes do not necessarily reflect bulk dopant activation, and highlight the need to combine complementary characterization techniques for a reliable assessment of MMLD processes.

## Graphical abstract

Mixed monolayer doping provides a controllable way to introduce phosphorus dopants into silicon. A gentle oxygen‐plasma treatment removes unwanted carbon while maintaining phosphorus on the surface. Following thermal diffusion, the treated silicon shows enhanced near‐surface n‐type behavior, demonstrating how carbon removal can tune molecular doping.

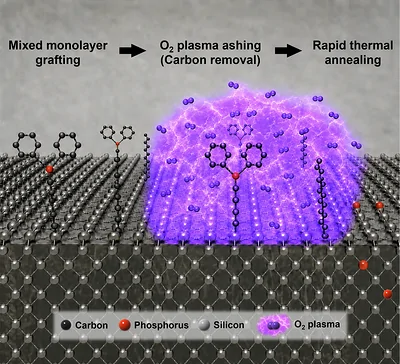

**Abbreviations**
ABAPE: allylboronic acid pinacol ester
AC: alternating current
ADP: allyldiphenylphosphine
AFM: atomic force microscopy
AM‐KPFM: amplitude‐modulation Kelvin probe force microscopy
BOX: buried oxide
DC: direct current
DPPA: diphenylphosphoryl azide
DVP: diethyl vinylphosphonate
HF: hydrofluoric acid
HOPG: highly oriented pyrolytic graphite
ICP: inductively coupled plasma
KPFM: Kelvin probe force microscopy
MID: mineral interface doping
MLCD: monolayer contact doping
MLD: monolayer doping
MMLD: mixed monolayer doping
PCB: printed circuit board
PECVD: plasma‐enhanced chemical vapor deposition
PLF: plasmon‐loss feature
PPD: polymer precision doping
RSF: relative sensitivity factor
RTA: rapid thermal annealing
SOI: silicon‐on‐insulator
SPV: surface photovoltage
TEOS: tetraethyl orthosilicate
THF: tetrahydrofuran
WF: work function
XPS: X‐ray photoelectron spectroscopy

## 1 Introduction

Ion doping is a key process in semiconductor technology, enabling controlled modification of the electrical properties of silicon through the intentional introduction of electrically active impurities. By incorporating group‐III or group‐V atoms into the silicon lattice, the carrier concentration can be tailored to produce p‐type or n‐type conductivity, respectively. Ion implantation is the industrial standard for introducing dopants into silicon with precise control of dopant dose and junction depth. However, in specific contexts such as ultra‐shallow junction formation, nanoscale devices, or extremely low dopant doses, ion implantation faces physical limitations. These include lattice damage caused by energetic ions, the need for postimplantation thermal annealing to electrically activate dopants, and the statistical nature of dopant placement, which becomes relevant as device dimensions shrink.

The concept of using molecular monolayers as a dopant source for ion diffusion was introduced in 2008^1^ and is known as monolayer doping (MLD). In this approach, molecules containing the desired dopant are typically grafted as a monolayer on the silicon surface [1, 2]. Following the removal of carbon by oxygen plasma ashing [3] or a low‐temperature oxidation [4] and the deposition of a SiO_2_ capping layer, the dopants are driven into the silicon lattice by rapid thermal annealing (RTA) [3, 5, 6]. However, recent studies have shown that this process sequence is not unique: phosphorus incorporation has also been demonstrated through oxide‐passivated or native‐oxide‐covered silicon surfaces, using tailored molecular precursors or oxide functionalization strategies [7, 8]. In some cases doping without the need of a capping layer using self‐capping precursor architectures has been demonstrated [9].

In MLD, the dopant dose is governed by the surface density and packing of the grafted molecules [3, 10]. As noted by some authors [10], achieving precise dose control in MLD remains challenging because the final dopant incorporation also depends on molecular decomposition kinetics and dopant diffusion during annealing. Nevertheless, MLD enables doping without causing crystal damage, in contrast to high‐energy ion implantation methods [2] and is therefore particularly attractive for the formation of ultra‐shallow [11] and conformal [12] doped regions on non‐flat surface features.

MLD has often been reported with polymeric molecules containing the dopant atom of interest such as phosphorus (P) [3, 5, 13]. This polymer‐based approach, often referred to as polymer precision doping (PPD) [3, 14], can involve molecules such as hyperbranched polyglycerols which can be as large as ∼19 nm in diameter [15].

The surface density and distribution of such macromolecular dopant‐containing species depends on steric effects [15] and polymerization parameters [14]. In some demonstrations, polymer‐based approaches like dopant‐containing block copolymers [16] or lithographic techniques such as nanoimprint lithography [17] have been used to obtain patterned doped regions.

These polymer‐based doping molecules however present several challenges. In particular, their high carbon content strongly reduces the activation rate of dopants such as phosphorus, that is, the fraction of dopant atoms that become electrically active after annealing, because carbon acts as an electron trap [18, 19]. In addition, these dopant‐containing polymers are not readily commercially available and require complex chemical synthesis processes, and the dose control can be affected by the thermal stability and degradation of the dopant‐carrying polymer during the annealing step [20]. Consequently, carbon removal is an important issue in polymer‐based doping. Recent publications in the field focus instead on improving the de‐carbonization step, either through thermal treatment [4] or oxygen plasma ashing [3, 6, 13, 15].

Carbon contamination should therefore be considered a process‐dependent limitation rather than an unavoidable feature of all molecular doping strategies. In addition to post‐grafting carbon removal treatments, carbon incorporation can be mitigated through the choice of the precursor and the process design itself. For instance, the functionalization of native or ultrathin SiO_2_ surfaces with phosphonic‐acid precursors enables the use of low‐carbon or carbon‐free dopant sources [7, 8]. In this strategy, if the selected molecule contains carbon, the oxide layer can also act as a barrier to carbon incorporation into silicon, since it has been reported that carbon‐containing residues remain preferentially on the SiO_2_ side of the Si/SiO_2_ interface during thermal annealing and can therefore be removed together with the oxide during the final HF treatment [8]. More broadly, carbon contamination can also be avoided by moving away from organic dopant precursors. Mineral interface doping (MID), for example, uses inorganic phosphate minerals as the dopant source, thereby avoiding carbon originating from organic precursors and relying on a different interface chemistry [21, 22].

Small molecules (around 1 nm^2^ molecular footprint), such as diethyl vinylphosphonate (DVP) [4], diphenylphosphoryl azide (DPPA) and allyldiphenylphosphine (ADP) [2, 23, 24] for phosphorus MLD, and allylboronic acid pinacol ester (ABAPE) [25] for boron MLD among others, have also been applied, demonstrating that smaller commercially available molecules can also be used for MLD. In addition, although O_2_ plasma (also known as ashing) has been used on PPD‐based systems, it has not yet been presented for MLD using small phosphorus‐containing molecules. These small‐molecule precursors inherently contain much less carbon than polymeric ones (typically ∼10–20 C atoms versus hundreds to thousands in polymeric chains), a feature that is favorable for achieving cleaner interfaces and reducing carbon‐related dopant deactivation, in line with the key role of carbon removal in MLD processes [4, 5].

Mixed monolayer doping (MMLD) has been proposed as a method to more precisely control the dopant dose in MLD by combining two molecules having the same binding mechanism which compete for attaching to silicon, and where one contains the doping atom of interest (e.g. P) while the other does not. Only a few studies [1, 10] have investigated the use of MMLD to control dopant dose.

In particular, previous studies [10] have shown that combining two molecules, such as DVP and 1‐undecene for phosphorus doping or ABAPE and 1‐undecene for boron doping allows dopant concentration tuning. Beyond dopant dose control, a key long‐term objective in silicon doping is the deterministic placement of individual dopant atoms, which is of particular interest for emerging applications such as solid‐state qubits [26].

To date, this challenge has been primarily addressed using ion implantation‐based approaches [27, 28, 29, 30]. In parallel, STM‐based hydrogen lithography has demonstrated the placement of individual P atoms with atomic‐scale (∼1 nm) precision [31]. Various strategies have been developed to adapt industry‐standard ion implantation tools for single‐ion control, including approaches based on active substrates, nanoapertures, nanostencils, or modified ion implantation systems [32, 33, 34, 35, 36, 37, 38]. These techniques rely on complex instrumentation and real‐time detection schemes, making them difficult to scale for large‐area device fabrication.

Molecular approaches such as MLD and MMLD provide an alternative route to introduce dopants into silicon based on surface chemistry rather than energetic ion insertion. Although oxide‐mediated and inorganic interface‐doping approaches offer alternative paths to mitigate carbon contamination and self‐capping precursor architectures can reduce dopant volatilization and avoid the need for an additional capping layer, hydrosilylated organic monolayers on H‐terminated Si remain attractive for MMLD because they offer the possibility to mix a dopant‐containing molecule with an undoped analogue to tune the nominal dopant dose. In this organic‐monolayer route, however, residual carbon from the grafted layer remains a relevant process limitation before dopant diffusion, while the phosphorus‐containing species must be retained. In particular, the role of carbon during the doping process has not been systematically investigated, although most reports on PPD show its impact on the dopant activation efficiency [18, 19]. Furthermore, a detailed study on how MMLD modifies the electronic structure of silicon (i.e. changes in the work function) has not been shown to date. In this context, Kelvin Probe Force Microscopy (KPFM) has previously been used for doping characterization [39, 40], including for monolayer contact doping (MLCD) [41].

In this work, we study silicon doping with MMLD and MLD using the small phosphorus‐containing molecule ADP, mixed with 1‐undecene to tune the nominal dopant dose. We evaluate the effect of O_2_ plasma (*ashing*) in removing carbon from the grafted monolayer prior to SiO_2_ capping and the impact it has on the subsequent electronic properties. The x‐ray photoelectron spectroscopy (XPS) was first used to determine the coverage of the silicon surface with ADP through the phosphorus signal and to assess the chemical changes induced on the grafted monolayer by O_2_ plasma. KPFM was then used to probe the near‐surface electronic response through work‐function measurements after dopant diffusion and capping‐layer removal. Finally, electrical measurements (four‐point probe and Hall effect) were performed to assess the contribution of electrically active dopants to the MMLD‐doped silicon, enabling a direct comparison between surface‐sensitive and bulk measurements.

**SCHEME 1:**
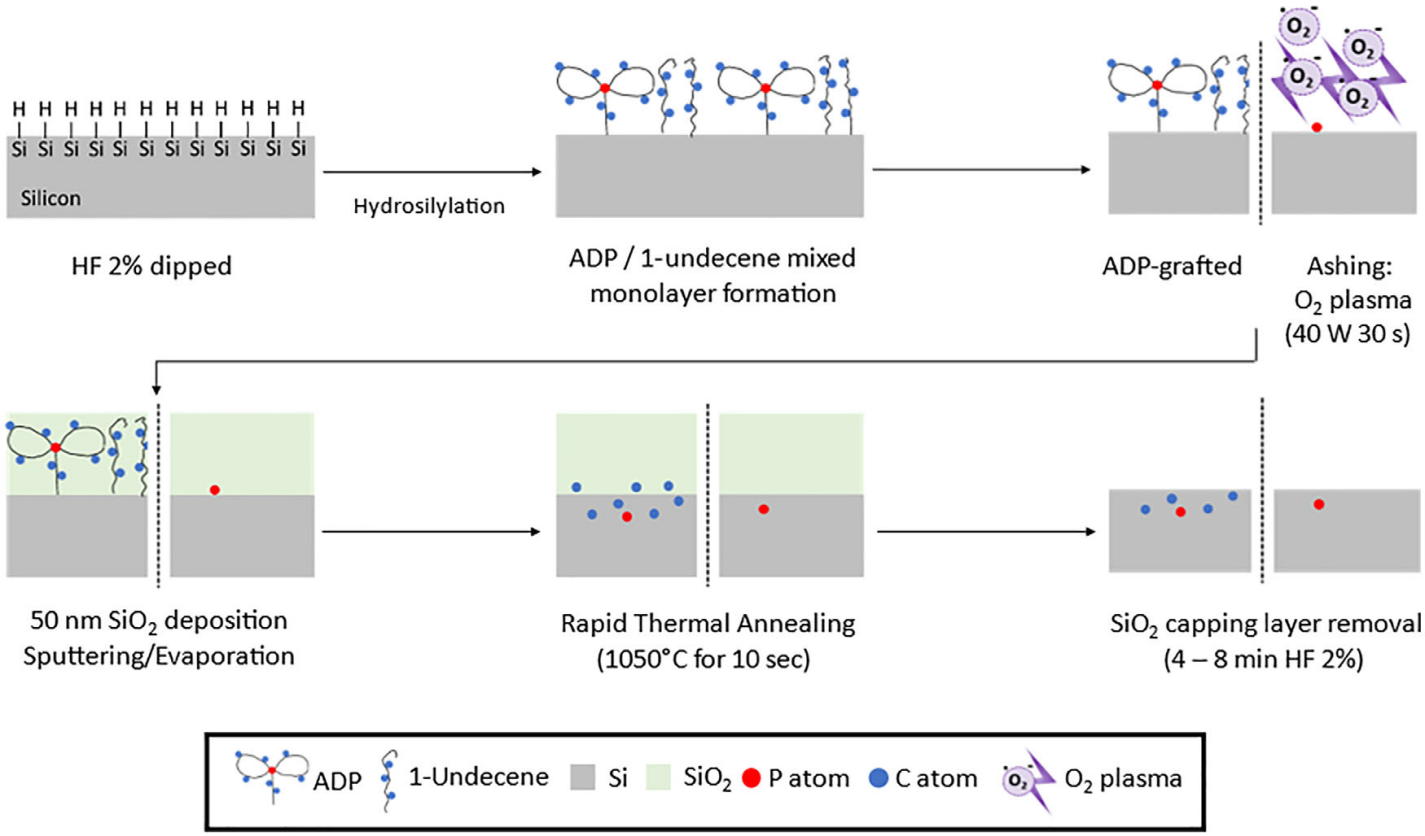
Schematic process flow of MMLD of silicon.

A schematic overview of the complete MMLD process flow used in this work is shown in Scheme 1.

Step‐by‐step schematic process flow for MMLD of silicon using ADP and 1‐undecene. From left to right and top to bottom: the Si substrate is cleaned and hydrogen‐terminated by a short HF dip (2%); mixed monolayers are grafted via hydrosilylation at 180°C for 5 h; pristine samples are left untreated, while ashed samples undergo O_2_ plasma (40 W, 30 s) to remove surface carbon before capping; a 50 nm SiO_2_ capping layer is deposited by sputtering or e‐beam evaporation; RTA (1050°C, 10 s) drives phosphorus into the Si lattice; the SiO_2_ layer is finally removed by HF dip (2%) (4–8 min).

## 2 Materials and Methods

### 2.1 Process Overview Description

The process starts by dipping a silicon chip into a diluted HF solution and therefore obtaining a hydrogen‐terminated silicon surface, which serves as substrate for molecular grafting. A mixed organic monolayer is then formed by the attachment of ADP and 1‐undecene molecules, with the relative surface composition determined by the ADP molar fraction and by the different reactivity and adsorption rates of each species, which compete for available surface sites during grafting.

After monolayer formation, two alternative processing routes are performed. In the first route, the grafted surface is directly encapsulated by a SiO_2_ capping layer, deposited either by electron‐beam evaporation or by sputtering. In the second route, the grafted monolayer is first exposed to an O_2_ plasma treatment prior to SiO_2_ deposition. In both cases, the SiO_2_ layer serves as a capping layer during the subsequent thermal treatment. The samples are then subjected to RTA, during which phosphorus from the monolayer‐derived source diffuses into the silicon substrate. After the annealing step, the SiO_2_ capping layer is removed, leaving a doped silicon surface. The two routes differ only by the presence or absence of the O_2_ plasma step prior to encapsulation, while the subsequent capping, annealing, and cap removal steps are identical. The O_2_ plasma treatment is introduced to selectively remove carbon from the monolayer while preserving phosphorus, with the hypothesis that reducing carbon‐related defects at the surface will enhance dopant activation, as carbon is known [18, 19] to act as an electron trap.

### 2.2 Molecule Dilution Preparation and Monolayer Formation

A silicon wafer (single side polished <100>p‐type (boron‐doped) Czochralski silicon wafers (Siegert Wafers, Germany), 525 ± 20 µm thick, 0.1 to 0.5 Ω·cm (corresponding to *N_A_* ∼4 × 10^17^ cm^−3^ to ∼3 × 10^16^ cm^−3^)) was diced into 1 cm dies. All glassware was cleaned in 3:1 H_2_SO_4_:H_2_O_2_ (piranha) rinsed with deionized and ultraclean water, dried and transferred into an argon‐filled glovebox (LabStar, MBraun, Germany, H_2_O < 0.5 ppm, O_2_< 0.5 ppm). Allyldiphenylphosphine (ADP, 98%, Lab Force) and 1‐undecene (97%, Sigma‐Aldrich) stock solutions (2% mol% in mesitylene 98%, Sigma‐Aldrich) were prepared in 100 mL double‐neck glass flasks. Dilutions of ADP were obtained by mixing weighted fractions of the two solutions. All solutions were degassed by 7 freeze‐pump‐thaw cycles (liquid N_2_ freeze, 70°C thaw), sealed and stored in the glovebox under argon. The silicon chips were cleaned by 2 min sonication in acetone and subsequently, sonicated for 2 min in a clean isopropanol solution and finally dried with nitrogen and transferred to the glovebox. Native oxide was removed by dipping the sample in 2% hydrofluoric acid (HF) for 17 s, followed by rinsing with inhibitor‐free tetrahydrofuran (THF), and drying under argon.

For surface functionalization, the freshly HF‐treated chips were placed inside a glass balloon containing the selected ADP dilution, connected to a condenser, and heated to 180°C for 5 h. After completion of the hydrosilylation reaction, the chips were immersed in a beaker containing THF and rinsed. The chips were dried under argon and stored individually in a piranha‐cleaned glass vial with a plastic cap, free of rubber or silicone joints to prevent outgassing.

### 2.3 O_2_ Plasma Treatment (*Ashing*) and Doping Process

Following monolayer grafting onto the silicon surface, the samples were split into two groups: (a) capped directly with SiO_2_ or (b) subjected to O_2_ plasma ashing, before the capping layer deposition, to remove the surface carbon present in the molecules forming the monolayer, while minimizing phosphorus loss. Samples that were not exposed to plasma will be referred to as *ADP‐grafted Si*, while those that were subjected to O_2_ plasma ashing will be referred to as *ADP‐grafted Si + O_2_ plasma*. This terminology is used consistently throughout the manuscript and figures.

For O_2_ plasma ashing, chips were mounted on dummy silicon wafers using Kapton tape inside a glovebox and then transferred to an inductively coupled plasma (ICP) system (Surface Technology Systems, STS). The O_2_ plasma ashing process to remove carbon was carried out in pure O_2_ (50 sccm ± 5%), at 20 mTorr with an RF coil power of 40 W and no platen bias to avoid direct ion bombardment of the silicon surface. The process duration was 30 s.

To assess how the capping method affects the doping, a 50 nm thick SiO_2_ layer was deposited either by sputtering or electron‐beam evaporation. Sputtering was performed in a Pfeiffer SPIDER 600 at a pressure of < 5 × 10^−7^ mbar with 1000 W RF power under Ar / O_2_ flow of 98/13 sccm for 3 min and 8 s. Evaporation was performed in a Leybold Optics LAB600H system at a pressure of 1.5 × 10^−6^ mbar using electron‐beam evaporation. Here, the chips were mounted on a dummy wafer using Kapton tape. During both deposition processes, a dummy wafer was included to allow direct SiO_2_ thickness measurement by spectroscopic ellipsometry (RC2, Woollam).

After capping, the samples underwent a RTA under nitrogen using a JetFirst 200 rapid thermal processor (Jiplec). The heating profile comprised two preheating steps (100°C for 10 s and 150°C for 30 s), followed by a rapid ramp (90°C s^−1^ up to 1050°C). The effective dwell time at 1050°C was about 10 s.

After annealing, the samples were returned to the argon‐filled glovebox, where the SiO_2_ capping layer was removed by etching in 2% HF. The required etching time was approximately 4 min for sputtered and 8 min for evaporated SiO_2_. Complete oxide removal for each case was visually confirmed by the characteristic hydrophobic behavior of H‐terminated silicon. The chips were rinsed with THF and dried with argon, stored in clean glass vials and sealed under vacuum.

### 2.4 Characterization

#### 2.4.1 XPS

Samples prepared for XPS were handled inside the glovebox as described above and mounted on clean microscope glass slides using low‐outgassing, chlorine‐free removable double‐sided tape. The slides with the mounted chips were placed in a Petri dish, sealed with Parafilm, and enclosed in vacuum bags. The slides were then quickly attached to the XPS sample holder and transferred to the load‐lock chamber under vacuum. Exposure to ambient air was kept below one minute to minimize contamination. All samples were analyzed within the same measurement session.

XPS measurements were performed using a Kratos Axis Supra Plus instrument equipped with a monochromated Al Kα x‐ray source (hν = 1486.6 eV, 500 mm Rowland circle). The emission current was 15 mA. All spectra were acquired in hybrid lens mode using a slot aperture, resulting in an analysis area of approximately 400 × 700 µm2. The samples were electrically insulated from the sample stage.

Samples with and without O_2_ plasma ashing, prepared as described above, were characterized by XPS. Each sample was analyzed at two distinct positions separated by a few millimeters. A survey spectrum (wide scan) was first recorded over the 0–1200 eV binding‐energy range with a step size of 1 eV, pass energy of 160 eV to identify all surface elements. Subsequently, high‐resolution scans of Si 2p, C 1s, O 1s, P 2p, and P 2s core‐level regions were acquired (pass energy of 40 eV, step size of 0.15 eV).

All spectra were processed using CasaXPS software. Binding‐energies were referenced at 284.8 eV to the CC/CH bond of the C 1s peak to correct for residual surface charging. Inelastic background contributions were removed using Shirley background subtraction to enable proper comparison between samples. Phosphorus quantification was based on the P 2s rather than the P 2p peak as the P 2p signal overlaps with a strong silicon plasmon‐loss feature (Si‐PLF) originating from the substrate. The P 2s region, although partially affected by the Si‐PLF, allows more reliable deconvolution. Gaussian–Lorentzian (GL) functions were used to separate the phosphorus peak from the Si‐PLF contribution. The shape of the Si‐PLF was evaluated by fitting its manifold on a reference sample not containing phosphorus. For visualization and quantification, the Si‐PLF manifold was subsequently subtracted from the Shirley‐corrected spectrum to isolate the phosphorus signal.

Elemental quantification for phosphorus, silicon, and carbon was performed by integrating the background‐corrected peak areas (using the deconvoluted phosphorus component) and applying the instrument‐specific relative sensitivity factors (RSFs).

#### 2.4.2 KPFM

All samples characterized by KPFM, except for some control samples, were measured after dopant diffusion and capping‐layer removal. All samples were etched in 2% HF for at least 4 min to remove both the SiO_2_ capping layer and the native oxide, rinsed with THF, and handled completely inside the glovebox. The samples were subsequently placed in argon‐filled glass vials, sealed in vacuum bags for transport, and introduced into a nitrogen glovebox housing the KPFM system (O_2_< 0.1 ppm, H_2_O < 0.1 ppm).

The KPFM measurements were performed using a Nanosurf FlexAFM connected to a lock‐in amplifier (HF2LI, Zurich Instruments) installed inside a nitrogen glovebox. AC and DC voltages were applied to the sample, while the conductive AFM tip was grounded. This configuration enabled the precise detection and compensation of electrostatic forces, allowing the contact potential difference (*V_CPD_*) to be determined quantitatively [42], which is directly related to the local surface work function (WF) and thus sensitive to changes in near‐surface doping.

Silicon cantilevers (*k* ≈ 48 N/m, ⌀_tip_ ≈ 20 nm) coated with a 25 nm PtIr_5_ conductive layer were used as probes (PPP‐NCLPt tips, Nanosensors). The measured resonance frequency of the first and second eigenmodes appeared near 170 kHz, and 1.05 MHz respectively.

Measurements were performed in single‐pass amplitude‐modulation KPFM (AM‐KPFM) mode. The first resonance mode maintained the cantilever oscillation in tapping mode to acquire the surface topography, while the second resonance mode was electrostatically excited by applying an AC bias *V_AC_* sin (ω*t*). Where *V_AC_* is the amplitude of the applied alternating voltage and ω = 2π*f*_2_ corresponds to the angular frequency of the second resonance mode (*f*_2_). The resulting oscillation amplitude at *f*_2_ was detected by the lock‐in amplifier and used to nullify the electrostatic force component, thereby determining the *V_CPD_* [42].

As the surfaces were structurally and electrically homogeneous, bias‐spectroscopy was mainly used. These measurements were performed by fixing the tip position above the sample and sweeping the DC bias voltage (*V_DC_*) applied between the tip and the sample. During the sweep, the cantilever electrostatic response was recorded using the lock‐in amplifier.

The resulting amplitude (*R*), measured by the lock‐in amplifier at the second resonance (*f_2_*), follows a linear dependence on *V_DC_*. The minimum (*R* = 0) corresponds to the contact potential difference (*V_CPD_*). The *V_CPD_* value was obtained by performing a linear fit on the in‐phase signal and extracting the *V_DC_* = *V_CPD_* at the x‐intercept, where *R* = 0. Slope and offset errors were acquired and used for the final error calculation.

Every two measurements, the tip WF was calibrated by measuring freshly cleaved highly oriented pyrolytic graphite (HOPG) which is known to have a WF of *Φ_HOPG_* = 4.6 eV [43]. Using this reference, the WF of the sample was determined according to:

(1) $$\phi_{Sample}=qV_{CPD}+\phi_{Tip}$$

where *V_CPD_* is the contact potential difference, ϕ _*Tip*_ the tip WF and *q* the elementary charge.

Each sample was measured at least at two microscopically different surface locations. The reported values were averaged arithmetically, and the uncertainty was calculated as the quadratic average of the individual errors.

#### 2.4.3 Four‐Point Probe and Hall Effect Measurements

All electrically characterized samples were fabricated on Silicon‐on‐Insulator (SOI) substrates (<100> p‐type (boron‐doped), device layer thickness 50 ± 10 nm, resistivity 8–12 Ω·cm; buried thermal oxide (BOX) thickness 400 nm ± 10%; handle layer thickness 725 ± 15 µm, resistivity 8–12 Ω·cm), following the same processing protocol described above. After removal of the capping layer, Ti/Au contacts (10 nm/150 nm) were defined by liftoff at the four corners of each die. Just before the metal deposition, the samples were dipped in 2% HF for 40 s to remove the native oxide and ensure direct metal–silicon contact.

The use of SOI substrates allows confinement of the electrically active region to a thickness comparable to the expected dopant diffusion depth. The diffusion depth is theoretically estimated to be around 50 nm, when considering the point at which the dopant concentration approaches the intrinsic carrier concentration of silicon (∼10^10^ cm^−3^). However, the majority of the dopant dose is estimated to be located within the first 15 nm approximately (see Section S4). In bulk Si wafers (∼525 µm), the contribution of the doped region to the overall conductance is negligible. By limiting the Si thickness to 50 nm, the impact of dopant incorporation is expected to become electrically measurable. The dies were wire‐bonded to a dedicated PCB enabling van der Pauw and Hall effect measurements.

Four‐point probe measurements were performed in van der Pauw geometry using a Keithley 2400 SourceMeter (Tektronix/Keithley Instruments). Current–voltage (*I*–*V*) sweeps were acquired in complementary contact configurations, and the corresponding resistances were extracted from linear fits of the *I*–*V* curves.

Hall measurements were performed in van der Pauw geometry using a Bruker 2T electromagnet to apply the magnetic field. A constant current of 10, 50, or 100 µA (adjusted according to the sample resistance to ensure operation within the voltage compliance limits of the instrument) was applied through the sample using the Keithley 2400 SourceMeter. The transverse Hall voltage was amplified using a Princeton Applied Research Model 5113 low‐noise preamplifier and recorded via a National Instruments data acquisition system. The Hall coefficient was extracted from the linear fit of the Hall voltage as a function of magnetic field.

Because MLD produces a depth‐dependent carrier distribution rather than a uniform conductive layer, the Hall results are reported as sheet carrier density (cm^−2^) rather than converted into an effective bulk carrier concentration. An estimation of the carrier mobility as extracted from the Hall measurements is provided in the Section S5.

A depth‐dependent multilayer model was used to account for the nonuniform carrier distribution by considering the dopant concentration profile across the device thickness and integrating its contribution to the Hall response. This analysis was used to assess the validity of the sheet approximation, with deviations remaining below ∼10% (see Section S2).

## 3 Results and Discussion

### 3.1 Surface Analysis: XPS

**FIGURE 1:**
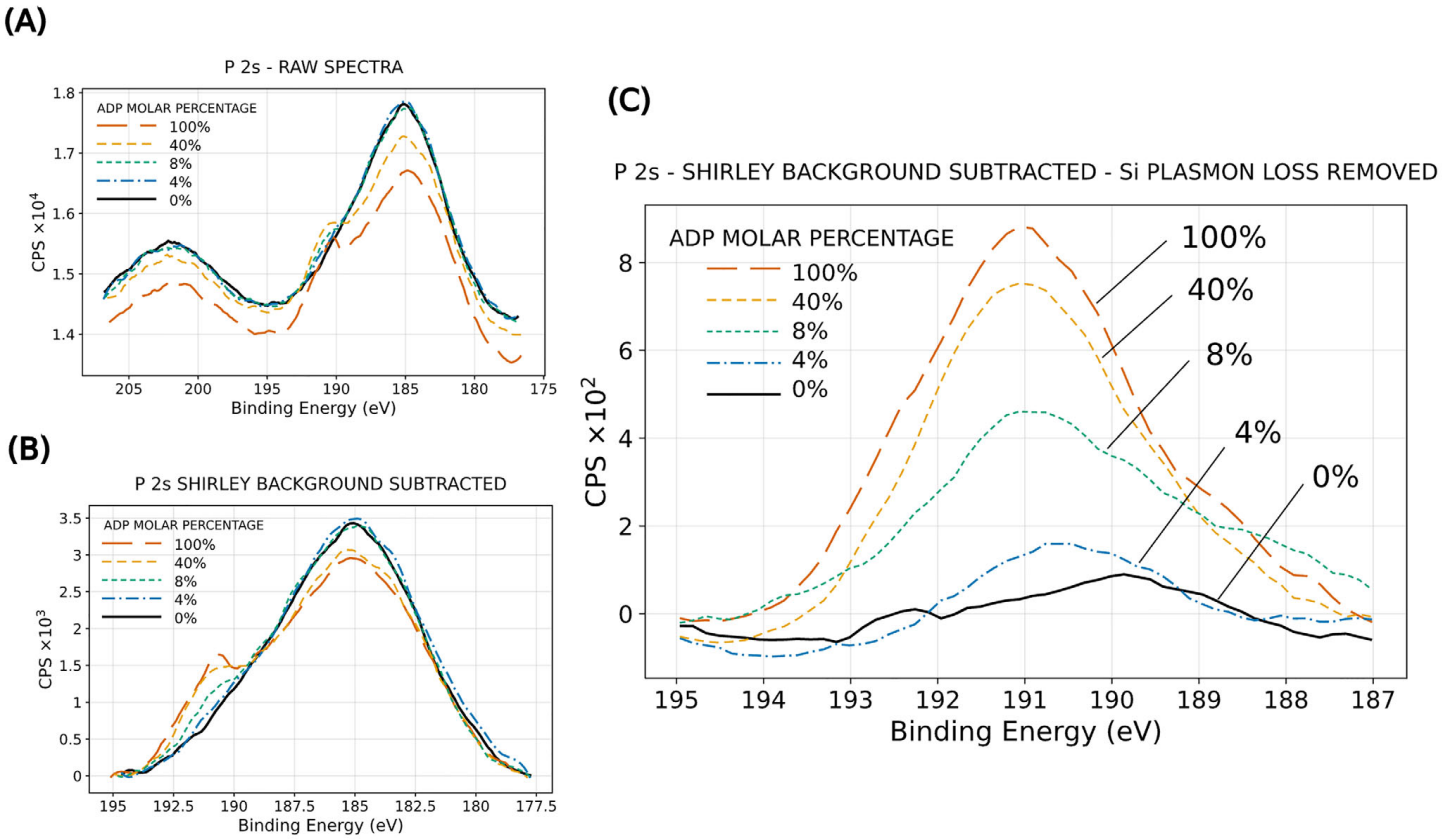
XPS P 2s spectra of the silicon surface after mixed monolayer formation with different ADP molar percentages. Spectra corresponding to 0%, 4%, 8%, 40%, and 100% ADP molar percentages are shown. (A) Raw XPS data of the P 2s region. (B) P 2s spectra after Shirley background subtraction. (C) P 2s spectra after Shirley background subtraction and removal of the Si plasmon‐loss feature (Si‐PLF) contribution.

Figure 1 shows the XPS P 2s spectra of silicon surfaces functionalized with mixed ADP/1‐undecene monolayers made with different ADP molar fractions. All measured samples only underwent the monolayer grafting step without any further processing (no capping and annealing). Figure 1A shows the raw data, Figure 1B shows the spectra after Shirley background subtraction (195 to 177.5 eV), Figure 1C, the spectra after both Shirley background subtraction and Silicon plasmon‐loss feature (Si‐PLF) removal. The Si‐PLF contribution was removed by Gaussian–Lorentzian (GL) deconvolution. The P 2s region contains a phosphorus‐related peak (∼195 to ∼189 eV) overlapping the Si‐PLF peak (∼195 to ∼177.5 eV).

Figure 1C shows a clear increase in the P 2s peak intensity at ∼191 eV with increasing ADP molar percentage. This confirms that the ADP molecules present in the different dilutions are covalently grafted onto the silicon surface by hydrosilylation reaction since the only possible source of phosphorus is the ADP molecule. This also indicates that 1‐undecene molecules occupy Si‐H sites, thereby limiting ADP grafting at low concentrations.

The Si‐PLF peak also changes in intensity with varying ADP molar fraction (Figure 1B) suggesting modifications in the plasmon‐loss response, likely related to the surface molecular coverage and composition, affecting the collective electron oscillations responsible for the plasmon‐loss features.

Subjecting the sample to O_2_ plasma ashing aims at removing carbon from the grafted monolayer while retaining as much phosphorus as possible, thereby improving dopant activation efficiency. This approach is inspired by the work [5, 6, 14] where ashing was successfully applied to phosphorus‐containing polymers used in PPD. Carbon removal is critical, as carbon diffusing into silicon can act as an electron trap, reducing the activation of phosphorus dopants.

**FIGURE 2:**
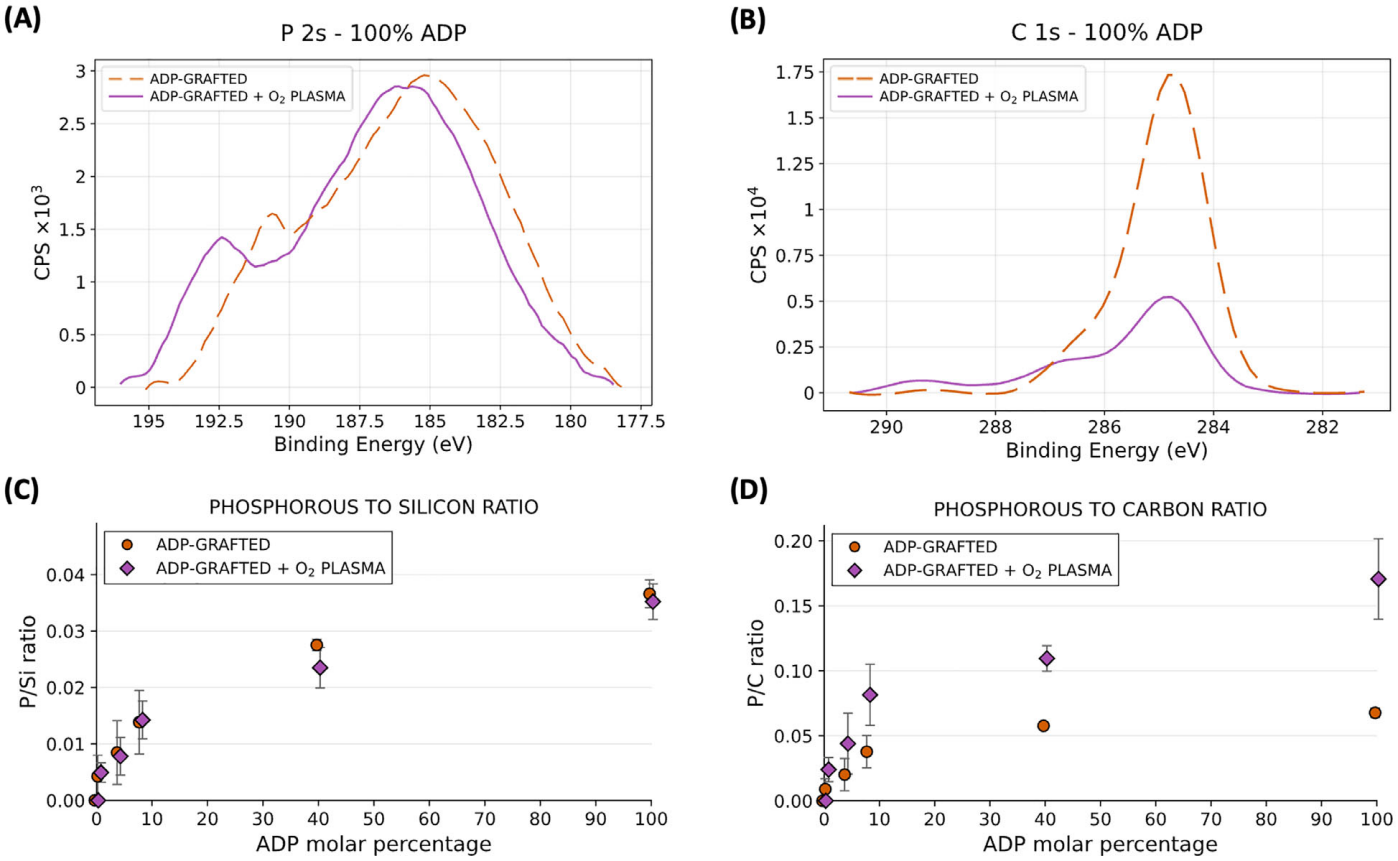
(A) Shirley‐background‐subtracted XPS P 2s spectra of silicon surfaces after monolayer formation with 100% ADP (ADP only, no 1‐undecene), comparing ADP‐grafted Si before and after O_2_ plasma ashing. (B) XPS C 1s spectra. (C) Evolution of the P/Si atomic ratio with the ADP molar percentage. (D) Evolution of the P/C atomic ratio with the ADP molar percentage.

Figure 2A compares the P 2s spectra of silicon surfaces after monolayer formation with ADP only, without any 1‐undecene (100% ADP molar percentage) before and after O_2_ plasma ashing. The untreated (ADP‐grafted) sample shows a P 2s peak around 191–189 eV, typically associated with P–C bonding and partially oxidized phosphorus species (between phosphine (P^3+^) and phosphate (P^5+^)), possibly caused by brief air exposure during transfer to the XPS chamber. All spectra were referenced to the C 1s carbon peak at 284.8 eV; therefore, small contributions from changes in the carbon environment cannot be fully excluded when comparing binding energies.

After O_2_ plasma ashing, the P 2s peak appears at higher binding energies (≈194–192 eV), consistent with phosphate groups (PO_4_3^−^).

Figure 2B shows the corresponding C 1s spectra. The C 1s peak, centered at 284.8 eV and corresponding to aliphatic carbon (also used for calibration), decreases significantly in intensity from about 1.75 × 10^4^ CPS to 0.5 × 10^4^ CPS after O_2_ plasma ashing confirming surface carbon removal. A small shoulder near 287 eV appears in the plasma‐treated sample, corresponding to oxidized carbon (C─O or C═O bonds), likely resulting from brief ambient exposure during sample loading into the plasma tool. Despite these minor oxidized species, the overall carbon content clearly decreases after O_2_ plasma treatment. Figures 2C and 2D show the P/Si and P/C atomic ratios as functions of ADP molar percentage. Each data point represents the mean of two measurements, with error bars corresponding to the standard deviation.

The P/Si ratio (Figure 2C) does not scale linearly with ADP molar percentage. This behavior is consistent with competitive surface binding between ADP and 1‐undecene during monolayer formation, as the two molecules compete for the available H–Si sites [10]. Both untreated and plasma‐treated samples exhibit the same qualitative trend, though plasma‐treated samples show slightly lower P/Si ratios at higher ADP percentages (40–100%), indicating a small but measurable phosphorus loss during the ashing step.

In contrast, the P/C ratio (Figure 2D) shows a similarly nonlinear dependence on the ADP molar percentage, but is systematically higher for plasma‐treated samples, especially at high ADP molar percentages. This demonstrates that the O_2_ plasma treatment removes carbon more efficiently than phosphorus, leading to a relative enrichment of phosphorus with respect to carbon in the monolayer‐derived layer. In other words, the plasma treatment preferentially reduces the carbon content while largely preserving the phosphorus content.

These results demonstrate that O_2_ plasma ashing can be successfully applied to small‐molecule‐based MLD to selectively remove carbon while largely retaining phosphorus. Since residual carbon in the near‐surface region is known to introduce electrically active defects and trap states that degrade dopant activation efficiency [18, 19], reducing the carbon content at the silicon interface improves the conditions for efficient phosphorus activation during the subsequent thermal diffusion step.

XPS measurements on silicon samples prepared with 100% ADP (ADP only, no 1‐undecene) after RTA and HF removal of the SiO_2_ capping layer were also performed using increased acquisition sensitivity; however, no phosphorus signal could be resolved. The corresponding Shirley‐background‐subtracted P 2s spectra are provided in the Section S7 (Figure S5). Based on the diffusion model, the expected near‐surface phosphorus concentration is on the order of 10^19^ cm^−3^, corresponding to approximately 0.02 at. %, which is below the reported XPS detection limit for phosphorus in silicon under Al Kα excitation (∼0.3–1 at.%) [44]. The overlap of the P 2s/P 2p regions with Si plasmon‐loss features further limits phosphorus detection. Post‐annealing electronic and electrical properties were therefore assessed using KPFM, four‐point probe, and Hall‐effect measurements.

**FIGURE 3:**
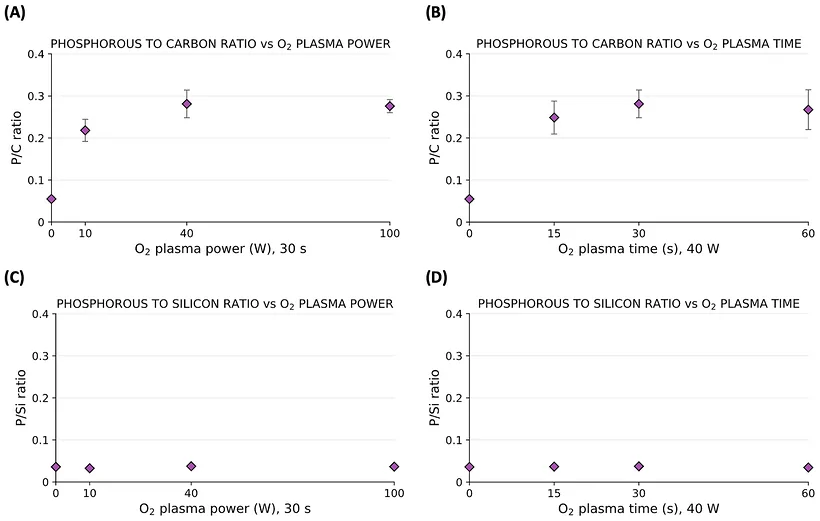
Evolution of the P/C and P/Si atomic ratios of a 100% ADP‐grafted silicon surface under different O_2_ plasma ashing conditions. The data point at 0 s or 0 W corresponds to the control sample, that is, the ADP‐grafted surface without O_2_ plasma treatment. (A) P/C ratio as a function of O_2_ plasma power at a fixed exposure time of 30 s. (B) P/C ratio as a function of O_2_ plasma exposure time at a fixed power of 40 W. (C) P/Si ratio as a function of O_2_ plasma power at a fixed exposure time of 30 s. (D) P/Si ratio as a function of O_2_ plasma exposure time at a fixed power of 40 W.

To further investigate the influence of the O_2_ plasma ashing step, the effect of plasma power and exposure time on the carbon removal efficiency was examined for a fixed monolayer composition. Figure 3 shows the evolution of the P/C and P/Si atomic ratios for a 100% ADP‐grafted silicon surface as a function of O_2_ plasma power (at a fixed exposure time of 30 s) and exposure time (at a fixed power of 40 W). Each data point represents the mean of three measurements, with error bars corresponding to the standard deviation.

In both cases, the P/C ratio (Figures 3A and 3B) increases rapidly at relatively low plasma power (up to approximately 40 W) or short exposure times (up to approximately 30 s), indicating efficient removal of carbon from the monolayer‐derived surface. Above these plasma conditions, the P/C ratio reaches a plateau and remains nearly constant upon further increasing either the plasma power (up to 100 W) or the exposure time (up to 60 s). This saturation behavior indicates that most of the removable carbon is already eliminated under relatively mild plasma conditions and suggests that increasing the plasma conditions beyond these thresholds does not lead to a significant loss of phosphorus.

In addition, the P/Si ratio (Figures 3C and 3D) remains essentially constant across the investigated plasma power and exposure time ranges. This indicates that no significant loss of phosphorus occurs during the plasma treatment, even under more aggressive conditions.

### 3.2 KPFM

**FIGURE 4:**
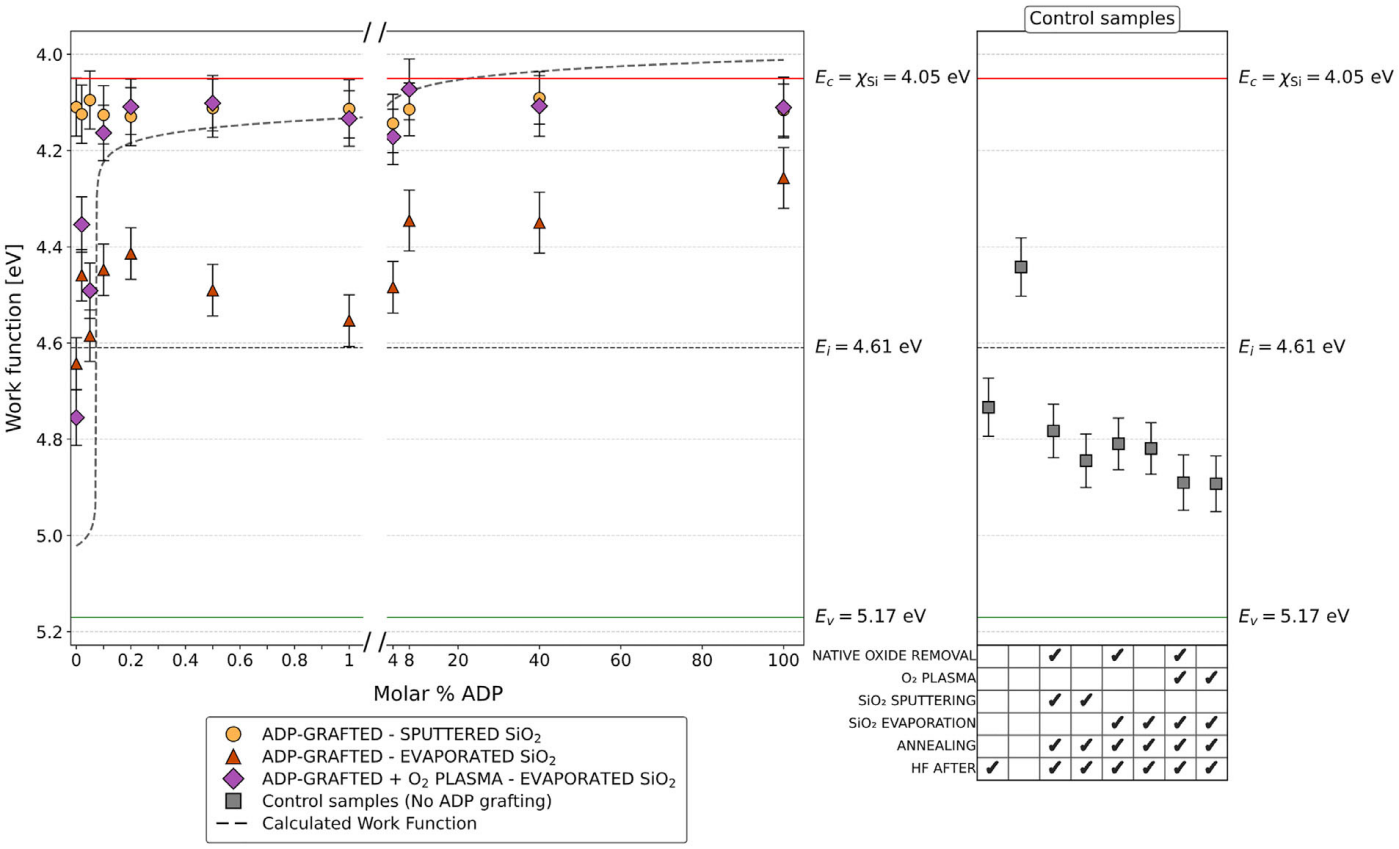
KPFM measurements presented as an energy‐band diagram of silicon showing the evolution of the WF of the silicon surface as a function of the ADP molar percentage, after SiO_2_ capping layer removal, for samples subjected to different treatments. Note that the y‐axis follows the conventional energy‐band representation, where higher energy levels are shown lower on the axis. The dashed line curve represents the calculated WF obtained from the limited‐source diffusion model described in the text. A refined dataset is included at low ADP molar fractions (0%–4%) with closely spaced values (0, 0.02, 0.05, 0.1, 0.2, 0.5%, 1%, and 4%) to capture the strong variation of the WF in this regime. The rightmost panel shows the WF values of the control samples, which were prepared without an ADP monolayer but subjected to the processing steps listed in the table below the plot.

Figure 4 shows how the WF of silicon varies with the percentage of ADP, presented as an energy‐band diagram. The upper and lower solid lines correspond to the silicon conduction band (CB) which also corresponds to the electron affinity of silicon (*E_c_* = *χ*_Si_ = 4.05 eV) and the valence band (VB) (*E_v_* = 5.17 eV) respectively. The horizontal dashed line represents the Fermi level of intrinsic silicon (*E_i_* = 4.61 eV). The dashed curve corresponds to the calculated WF obtained from the model described later in the text. Further details of the model and underlying assumptions are provided in the Section S1.

KPFM measurements were performed on silicon surfaces after SiO_2_ capping layer removal, as described in the Materials and Methods section. The results compare samples prepared under different conditions, including ADP‐grafted Si, ADP‐grafted Si + O_2_ plasma, and different SiO_2_ capping deposition methods (evaporation or sputtering).

To resolve the low‐concentration range, a set of samples with low ADP molar fractions (0, 0.02, 0.05, 0.1, 0.2, 0.5, 1 and 4%) was analyzed. The high‐concentration data points (8%, 40% and 100%) were taken from another batch, processed using the same fabrication protocol.

Each sample was measured at two different surface positions. Each point in Figure 4 represents the average WF of the two positions, and the error bars represent the quadratic average of the individual errors.

Control samples have a WF between ∼4.6 and ∼4.9 eV, below the mid‐gap value, which is consistent with p‐type doping. The measured values are lower than expected for boron doped silicon with nominally *ρ* = 0.1 to 0.5 Ω·cm (corresponding to an acceptor concentration *N_A_* = 3 × 10^16^ to 4 × 10^17^ cm^−3^), likely due to surface band bending effects that reduce the apparent WF [45]. Under flat‐band conditions, the WF for a p‐type semiconductor is:

(2) $$\Phi_{Sip-type}=\chi +\frac{E_{g}}{2}+k_{B}T\ln\left(\frac{N_{A}}{n_{i}}\right)$$

With the electron affinity of silicon (*χ* = 4.05 eV), the silicon band gap energy (*E_g_* = 1.12 eV), the Boltzmann constant (*k_B_*) and the absolute temperature (*T*) product at 300 K (*k_B_T* = 0.02585 eV), the intrinsic carrier concentration in silicon (*n_i_* = 1.45 × 10^10^ cm^−3^) and the acceptor concentration in cm^−3^ (*N_A_*).

Using Equation (2), we calculated WF values of 4.99–5.06 eV for the bare boron doped silicon. Taking our measured WF values into account, this results in surface band bending of 0.09–0.46 eV. This downward band bending is characteristic of p‐type Si (100) surfaces and is caused by surface states introducing positive charges to the surface and attracting electrons, thereby shifting the surface Fermi level toward the CB. The untreated control sample (with native oxide) exhibits a lower WF of around 4.45 eV due to enhanced band bending caused by fixed charges within the native oxide and or at the interface. All other control and functionalized samples were subjected to a brief HF dip prior to the KPFM measurement in order to eliminate the oxide layer and ensure consistent Si surface conditions. Previous studies [39, 42] using Kelvin probe and surface photovoltage (SPV) measurements on HF‐treated silicon have confirmed this asymmetry: p‐Si (001) exhibits significant band bending, with reported SPV values on the order of ∼0.4–0.5 V and built‐in potentials around ∼0.6 eV, whereas n‐Si (001) shows a negligible response. This is consistent with the dominant role of positive surface charges in p‐type materials.

For the mixed monolayer samples containing different ADP molar fractions, all measured WFs lie below the intrinsic level of silicon (4.61 eV), indicating n‐type behavior, except for the 0% ADP samples (only 1‐undecene grafting i.e., without any phosphorus‐containing molecules) capped by e‐beam evaporated SiO_2_, which exhibit WFs around 4.7 eV, consistent with the p‐type substrate and in good agreement with the control samples. The WF decreases rapidly between 0% and ∼0.2% ADP and then reaches a plateau close to the conduction‐band edge, remaining nearly constant up to 100% ADP. This demonstrates that even very small ADP fractions already lead to a strong modification of the near‐surface electronic properties.

A markedly different behavior is observed for samples capped by sputtered SiO_2_. All sputtered samples, including the 0% ADP sample, exhibit a strongly pinned WF around ∼4.1 eV, close to the conduction‐band edge, independently of the ADP molar fraction. This suggests that, for these samples, the measured WF is not governed by the dopant concentration but rather by surface related effects.

Possible explanations include fixed charges induced during the sputtering process, plasma‐induced surface states, or carbon‐related defects at the Si interface, which could introduce a high density of interface states and pin the surface Fermi level. To further investigate this point, preliminary SPV measurements were performed under 555 nm laser illumination with our KPFM system. However, no detectable WF change was observed, leaving the physical origin of this pinning effect unresolved. As these observations are based on a single SPV measurement per sample, they should not be considered a strong argument. Further investigation will therefore be required to distinguish between true doping effects and surface‐related electronic phenomena in these samples.

The different processing conditions tested significantly affect the WF. In particular, the O_2_‐plasma‐treated samples capped by evaporated SiO_2_ exhibit systematically lower WFs than samples capped by evaporation without plasma, indicating stronger apparent n‐type behavior. Because the sputtered samples are dominated by strong WF pinning, the evaporation series (with vs without O_2_ plasma) provides the clearest basis for comparing dopant activation.

To evaluate the doping quantitatively, KPFM results were compared to calculated WFs for each ADP molar percentage. The calculations assumed, as previously reported [46], an ADP surface footprint of 1.1 × 0.8 nm^2^, with dopant diffusion following Fick's second law for a limited‐source profile, where all phosphorus atoms initially present at the surface diffuse into the silicon lattice during annealing. Since KPFM probes surface electronic properties, we assumed that the effective dopant concentration for WF calculation corresponds to the first 2 nm of the diffusion profile. Under flat‐band conditions, the WF of an n‐type semiconductor differs from that of a p‐type semiconductor only by the sign of the logarithmic term:

(3) $$\Phi_{Sin-type}=\chi +\frac{E_{g}}{2}-k_{B}Tln\left(\frac{N_{D}}{n_{i}}\right)$$

The only difference is the donor concentration (*N_D_*) in cm^−^^3^ and the sign in front of *k_B_*.

For the 0% ADP case, no phosphorus is present on the surface; therefore, the WF corresponds to that of the p‐type substrate. To simplify the calculation, the midpoint of the manufacturer's specified acceptor concentration range was used, *N_A_* = 1.20 × 10^17^ cm^−3^.

For samples containing ADP, the introduction of phosphorus donors progressively compensates the initial p‐type doping. Therefore, the effective doping concentration governing the Fermi‐level position was taken as the net dopant concentration, *N_eff_* = |*N_D_*‐*N_A_* |. When *N_D_* <*N_A_*, the material remains effectively p‐type and Equation (2) is used with $N_{A}^{eff}=N_{A}-N_{D}$, while for *N_D_* >*N_A_* the material becomes n‐type and Equation (3) is used with $N_{D}^{eff}=N_{D}-N_{A}$. The resulting calculated WF is overlaid as a dashed line in Figure 4 and directly compared with the experimental data.

The compensation model predicts a very sharp transition of the WF when the donor concentration approaches the initial acceptor concentration (*N_D_*  ≈  *N_A_*), corresponding in our case to an ADP molar fraction of approximately 0.07%. However, such an abrupt transition is not observed experimentally. Instead, in the low‐ADP concentration region (≤ 0.05%), the measured WF evolves more smoothly and shows significant deviations from the idealized model prediction. This suggests that, in this concentration range, the KPFM response is not only dominated by the actual bulk dopant concentration but also surface effects.

The control samples show WFs in good agreement with the calculated values. In contrast, the 0% ADP sample capped by sputtered SiO_2_ exhibits a much lower WF (∼4.1 eV), despite the absence of phosphorus, strongly suggesting that in this case the measured WF is likely dominated by surface effects rather than by bulk doping as previously discussed.

For ADP concentrations ≥ 0.1%, good agreement between experiment and calculation is observed for samples that are likely not dominated by strong surface pinning. In particular, the O_2_‐plasma‐treated samples capped by evaporated SiO_2_ follow well the calculated trend. Samples capped by evaporation without plasma systematically show higher WFs, indicating weaker n‐type activation, likely related to residual carbon. In contrast, sputtered samples do not follow the calculated trend due to the strong pinning effects discussed above.

At higher ADP concentrations (40% and 100%), the calculated WFs exceed the conduction‐band minimum, corresponding to degenerate doping levels. In contrast, the experimental WF values of the O_2_ plasma‐treated samples remain close to the CB edge but do not exceed it. Although sputtered samples also appear close to the CB edge, this behavior is dominated by strong surface pinning and does not reflect the actual bulk doping level.

This discrepancy between calculations and experiments may indicate several non‐idealities. First, if many dopants are introduced into the silicon, not all of them contribute to free carriers, some are electrically inactive (trapped in non‐substitutional lattice sites or bonded to other atoms such as carbon or oxygen), resulting in an overestimated effective carrier density. Second, the model used in Equation (3) assumes both a high incorporation efficiency and 100% dopant activation. Third, phosphorus desorption during capping and annealing is not considered, leading to further overestimation of the calculated WF, which becomes more pronounced at high ADP concentrations. Additionally, negative surface charges associated with the organic monolayer or silicon‐molecule interaction could induce upward band bending, which would increase the apparent WF and partially counteract the expected reduction in WF associated with n‐type doping. Evaporated samples without O_2_ plasma deviate substantially from the calculations, suggesting reduced dopant activation, consistent with residual carbon‐related effects.

Overall, these results show that the measured WF is not only determined by the dopant dose, but results from a competition between bulk doping and surface‐related effects. In particular, strong surface band bending can dominate the KPFM response, as observed for all sputtered samples, independently of the ADP molar fraction.

The O_2_‐plasma‐treated samples capped by evaporated SiO_2_ follow the calculated trend most closely, which is consistent with improved interface cleanliness and reduced carbon‐related defects prior to dopant diffusion. In contrast, samples capped by evaporated SiO_2_ without plasma show systematically higher WFs, indicating weaker n‐type activation likely due to residual carbon. For sputtered samples, the strong pinning of the WF near ∼4.1 eV indicates that the surface‐related effects introduced by the sputtering process likely dominate the electronic response and mask the actual bulk doping level, so that no direct conclusion on dopant activation efficiency can be drawn from these samples.

The present comparison was restricted to evaporated and sputtered SiO_2_ caps in order to assess two capping routes while avoiding additional precursor chemistry during the capping step. However, SiO_2_ caps deposited by plasma‐enhanced chemical vapor deposition (PECVD) using tetraethyl orthosilicate (TEOS) have also been successfully used in device‐oriented MLD processes [47, 48]. Therefore, a systematic comparison of different SiO_2_ capping methods, including PECVD/TEOS‐deposited SiO_2_ caps, would be a valuable direction for future work to assess device‐process integration.

### 3.3 Electrical Measurements: Four‐Point Probe and Hall Effect Measurements

To complement the surface‐sensitive XPS and KPFM analyses, electrical transport measurements were performed on SOI samples in van der Pauw geometry in order to probe the electrical transport properties, including sheet resistance and carrier density, beyond the topmost surface region sensed by KPFM. Four‐point probe measurements were carried out using a square geometry, with one metal contact at each corner of the chip. A current was injected between two adjacent contacts while the voltage drop was measured across the opposite pair, yielding current–voltage (*I*–*V*) curves for the two complementary configurations. The resistance for each configuration was extracted from the slope of the corresponding I–V curve, and the sheet resistance was then determined using the general van der Pauw formalism:

(4) $$e^{\left(-\frac{\pi R_{AB,CD}}{R_{S}}\right)}+e^{\left(-\frac{\pi R_{BC,DA}}{R_{S}}\right)}=1$$

where *R_S_* corresponds to the sheet resistance and *R_AB, CD_*, *R_BC, DA_* to the resistance measured in two complementary configurations.

**FIGURE 5:**
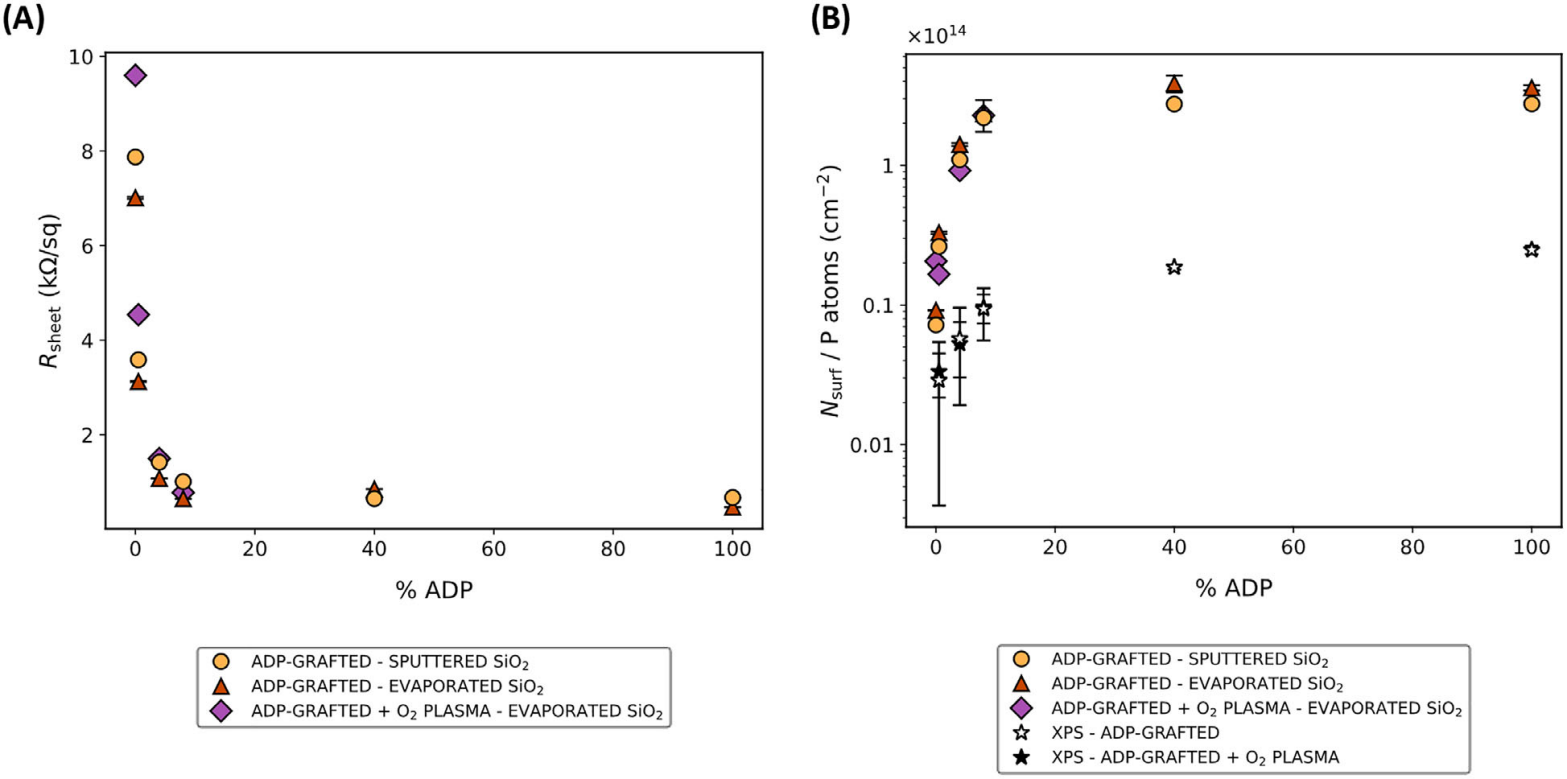
(A) Evolution of the measured sheet resistance, *R*_sheet_, as a function of ADP molar percentage for samples processed under different capping and surface‐treatment conditions. (B) Evolution of the electrically active sheet carrier density extracted from Hall‐effect measurements and the phosphorus surface density estimated from XPS (uncorrected for attenuation effects) as a function of ADP molar percentage for samples processed with and without O_2_ plasma ashing. Error bars on the XPS‐derived values correspond to the standard deviation of the measured P/Si ratio.

Samples with different ADP molar percentages (0%, 0.5%, 4%, 8%, 40%, 100%) and processing conditions were measured. The extracted sheet resistance values are plotted as a function of ADP molar fraction in Figure 5A. The error bars represent the propagated uncertainty, including both the standard deviation between complementary configurations and the fitting error associated with the slope extraction of the *I–V* curves.

As shown in Figure 5A, the sheet resistance decreases sharply with increasing ADP molar fraction. The 0% ADP samples (i.e., 100% 1‐undecene) exhibit resistances on the order of ∼10 kΩ/sq, while samples with ≥ 8% ADP reach values below ∼1 kΩ/sq and remain relatively constant up to 100% ADP. This behavior indicates that increasing the fraction of the phosphorus‐containing molecule in the mixed monolayer leads to a significant enhancement of electrical conductivity after dopant diffusion and activation.

This saturation is consistent with the evolution of the P/Si ratio observed by XPS (Figure 2C) which indicates that the final surface composition does not directly reflect the molar ratio in solution due to competitive surface binding between ADP and 1‐undecene molecules during monolayer formation. As a result, the maximum achievable dopant density at the interface is limited, as previously reported for MLD and MMLD systems [1, 3, 10]. However, it cannot be explained only by this effect, but rather suggests a more complex interplay between dopant concentration, carrier mobility, and dopant activation within the silicon lattice.

At low ADP molar fractions, the introduction of phosphorus donors leads to a strong increase in electrical conductivity, as carriers are added in a regime where impurity scattering is limited and mobility is therefore expected to be relatively high [49]. In this regime, both the sheet resistance and the carrier density change rapidly with increasing dopant dose.

As the dopant concentration increases, the electrical response evolves more gradually. This behavior can be attributed to a higher number of defects introduced by the dopant atoms, which reduce carrier mobility and therefore limit the increase in conductivity despite the higher dopant dose.

At higher ADP molar fractions, the saturation of both sheet resistance and carrier density indicates that not all incorporated phosphorus atoms become electrically active. This limitation is likely driven by a combination of incomplete dopant activation, trap states, and dopant‐dopant interactions such as the formation of clusters, which result in a reduction of the activation efficiency. Consequently, further increases in dopant dose do not lead to higher carrier densities [18, 19].

Control samples without any monolayer were also prepared; however, their resistance was too high to be reliably measured under the present conditions due to the intrinsic resistivity of the SOI device layer. The comparatively lower resistance observed in the 0% ADP samples therefore suggests the presence of a background contribution associated either with the fabrication process itself or with the monolayer. The origin of this contribution remains unclear and requires further investigation.

Overall, the strong dependence of the sheet resistance on the ADP molar fraction demonstrates the capability of tuning the electrically active dopant dose through precise control of the mixed monolayer composition.

On the other hand, no clear difference is observed when comparing samples treated with O_2_ plasma to those without plasma treatment. As previously discussed, carbon is expected to contribute to the deactivation of phosphorus dopants, likely through the formation of C_i–_P_s_ pairs with multiple deep energy levels [19]. However, despite the effectiveness of O_2_ plasma in removing carbon as evidenced by XPS, no significant variation is observed in the electrical measurements. This suggests that carbon‐related dopant deactivation is not sufficiently extended in depth to significantly impact the overall carrier transport despite possible surface‐localized deactivation.

This apparent discrepancy can be attributed to the different probing depths of the techniques. While KPFM is sensitive to the near‐surface region (typically within the first 2–3 nm), electrical transport measurements probe the integrated response across the conductive layer. Since carbon has been reported [50] to have its diffusion limited to approximately 2–3 nm, its impact on dopant deactivation is expected to be more pronounced in surface‐sensitive measurements such as KPFM than in bulk‐sensitive electrical measurements. Although carbon‐related defects can locally deactivate phosphorus dopants, the thickness of the affected region represents only a small fraction (about 2 nm) of the total conductive layer (about 15 nm (see Figure S2)), and therefore its contribution to the overall electrical transport seems to be negligible.

In addition to four‐point probe measurements, the same samples were further characterized by Hall‐effect measurements in a van der Pauw configuration. A constant current was applied between two opposite contacts, while the transverse voltage was measured across the remaining contacts under a perpendicular magnetic field. The Hall voltage was recorded as a function of the magnetic field, and the Hall coefficient (*R_H_*) was extracted from the slope of the linear *V_H_‐B* response:

(5) $$R_{H}=\left(\frac{1}{I}\right)\left(\frac{dV_{H}}{dB}\right)$$

where *I* is the injected current and $\frac{dV_{H}}{dB}$, the extracted slope.

The corresponding sheet carrier density (*n_s_*) was calculated as:

(6) $$n_{S}=\frac{1}{qR_{H}}$$

where *q* is the elementary charge.

Equation (6) assumes a uniform carrier distribution across the conductive layer. However, given the surface‐limited nature of the doping process, the actual dopant profile is expected to be depth‐dependent. To assess the validity of this approximation, a depth‐dependent transport model based on a multilayer Hall formalism was implemented (see Section S2), accounting for variations in both carrier concentration and mobility with depth. The comparison indicates that the deviation in the computation of *n_s_* introduced by the uniform approximation remains below ∼10%, supporting the validity of the simplified uniform approximation and in agreement with previously reported analyses for molecularly doped silicon systems [19].

Given the depth‐dependent dopant distribution, which decreases from the surface into the bulk due to the limited‐source diffusion process, the definition of a volumetric carrier concentration (cm^−3^) is not physically meaningful in this system. Therefore, the results are expressed in terms of sheet carrier density (cm^−2^). For the same reason, the extraction of carrier mobility is not reported, as it would require assuming a uniform carrier distribution throughout the depth of the sample.

Since the Hall measurements provide the sheet carrier density, and these carriers are expected to originate from phosphorus donors, this quantity can be compared with the phosphorus surface density estimated from the XPS measurements (Figure 2C). A direct conversion of the measured P/Si ratio into a surface density can be performed by multiplying it by the surface atomic density of Si (100) (∼6.78 × 10^14^ atoms/cm^2^). However, such a direct approach is oversimplified because the Si 2p signal in XPS is not restricted to the first atomic layer, but also includes attenuated contributions from silicon atoms located below the surface, leading to an underestimation of the true phosphorus surface density by a factor that is difficult to determine precisely but is expected to be on the order of 6 to 10. (See Section S3 for details).

As a result, the raw P/Si ratio does not directly represent the fraction of surface Si atoms bonded to phosphorus, and therefore the phosphorus surface densities estimated from XPS should therefore be interpreted as lower‐bound values. The XPS‐derived values shown in Figure 5B are presented without applying any attenuation correction and should be interpreted as lower‐bound estimates of the phosphorus surface density. While these values capture the relative trends with ADP molar fraction, they underestimate the absolute dopant density, and applying a correction factor would bring the XPS‐ and Hall‐derived sheet carrier density values closer to each other. The estimation of the phosphorus surface density from XPS, including correction factors derived from attenuation effects, is detailed in the Section S3). The sheet carrier density was measured for each sample and is shown on a logarithmic scale in Figure 5B, together with the phosphorus surface density estimated from XPS. The carrier density increases with the ADP molar fraction following the same trend as the sheet resistance (Figure 5A). The carrier concentration increases from ∼2 × 10^13^ cm^−2^ at 0% ADP, to ∼4 × 10^14^ cm^−2^ where it saturates at higher ADP percentages (40%–100%). Also, similarly to what was already observed, and in contrast to the results measured by KPFM, no significant difference in the number of active carriers was observed between the samples treated with O_2_ plasma and not treated with O_2_ plasma. As previously discussed, this would need some further study but may be related to the short diffusion depth of carbon which makes more evident the effect of carbon on the dopant activation when measured on the surface.

Hall‐derived carrier densities and XPS‐derived phosphorus surface densities follow a similar trend. The XPS‐derived values systematically lie below the Hall‐derived ones, typically by a factor of a few (up to about one order of magnitude for some samples). This discrepancy may arise from several factors: (i) as discussed previously, a correction factor needs to be applied to the phosphorus contribution, or the fact that (ii) additional carriers may be introduced by the process itself (as suggested by the nonzero carrier density observed in 0% ADP samples) impacting the Hall data.

A more quantitative comparison between Hall‐derived carrier densities and XPS‐derived phosphorus surface densities is provided in the Section S6, where both quantities are shown to follow a clear positive correlation and lie within the same order of magnitude.

## 4 Conclusion

This work demonstrates a viable route toward controllable silicon n‐type doping using mixed‐monolayer doping (MMLD) and provides new insights into the role of O_2_ plasma‐assisted carbon removal by combining surface‐sensitive and electrical characterization techniques.

A first key result is that O_2_ plasma ashing, previously mainly applied to polymer‐based dopant sources, is also highly effective for small‐molecule‐based monolayers with ∼1 nm^2^ footprint. XPS confirms that the plasma step selectively removes carbon while largely preserving phosphorus, and KPFM reveals a systematic reduction of the WF with increasing ADP molar fraction, with a stronger decrease after O_2_ plasma treatment, consistent with enhanced n‐type activation at the surface. These results indicate that O_2_ plasma ashing can be used as a post‐grafting step to reduce the carbon content of small‐molecule MMLD monolayers while preserving phosphorus on the surface, leading to measurable changes in the near‐surface electronic response.

By combining KPFM with electrical measurements, this work also shows that the electronic response of MMLD of silicon cannot be interpreted solely from surface‐sensitive techniques. While KPFM indicates a clear enhancement of n‐type behavior after O_2_ plasma treatment, no significant difference is observed in either sheet resistance or Hall‐derived carrier density between O_2_ plasma‐treated and untreated samples. This discrepancy highlights a fundamental difference between surface‐sensitive and bulk‐integrated measurements.

This difference can be explained by the limited diffusion depth of carbon, which has been reported [50] to be confined to approximately 2–3 nm. As a result, carbon‐related defects predominantly affect the near‐surface region probed by KPFM, while their contribution to dopant deactivation remains spatially confined and represents only a minor fraction of the total conductive layer. Consequently, their impact on the overall electrical transport is negligible, which is dominated by the deeper region where phosphorus has diffused and remains electrically active.

At the same time, this study clearly shows that the KPFM‐measured WF is not only determined by the dopant dose, but results from a competition between bulk doping and surface‐related electronic effects. In particular, the strong surface band bending observed for all sputtered samples indicates that surface‐related effects introduced during the capping process can dominate the KPFM response and mask the actual bulk doping level, preventing any quantitative conclusion on dopant activation from being drawn in this case.

In contrast, samples treated by O_2_ plasma and capped by evaporated SiO_2_ follow the calculated trend most closely, consistent with a more defect‐free silicon surface and reduced carbon‐related defects. This processing route therefore provides the clearest conditions in this study to assess and control the near‐surface electronic response of MMLD‐doped silicon using KPFM.

Electrical measurements further confirm that the carrier density increases with ADP molar fraction and saturates at high concentrations. This behavior is consistent with the trend observed in the P/Si ratio extracted from XPS measurements, indicating that the final surface composition does not directly reflect the molar ratio in solution. Instead, it suggests differences in surface binding between the molecular species during monolayer formation. Also, this saturation likely reflects a combination of these surface effects and intrinsic limitations in dopant incorporation and/or activation efficiency during diffusion.

Overall, this work presents plasma‐assisted MMLD with small molecules as a promising and tunable approach for silicon doping while also demonstrating that the understanding of dopant activation requires combining surface‐sensitive and electrical transport measurements. The results show that changes in the surface WF do not necessarily translate directly into increased electrically active carrier density, highlighting the fundamental difference between surface‐sensitive and bulk‐integrated measurements.

These findings emphasize that the choice of measurement approach for MLD‐doped samples becomes critical, particularly in the near‐surface doping, which is especially relevant for emerging applications such as quantum devices.

## Author Contributions

Pol Torres‐Vila contributed to conceptualization, experimental design and execution, sample characterization, data analysis, and writing of the manuscript. Mounir Mensi contributed to XPS measurements and analysis. Thilo Glatzel contributed to KPFM measurements and analysis. Giovanni Boero contributed to the four‐point probe and Hall effect measurements. Juergen Brugger and Arnaud Bertsch supervised the project. All authors reviewed and approved the final version of the manuscript.

## Funding

This work was supported by the NFFA‐Europe Pilot Project (Grant agreement No. 101007417) and the Swiss National Science Foundation (SNSF) (Grant No. 10.002.312).

## Conflicts of Interest

The authors declare no conflicts of interest.

## Supporting information




**Supporting File**: smtd70970‐sup‐0001‐SuppMat.pdf

## Data Availability

The data that support the findings of this study are available from the corresponding author upon reasonable request.
