# Supplementary material for: Impact of Plasma Ashing on Mixed Monolayer Doping of Silicon
Source: Small Methods. 2026 Aug 17;10(18):e70970. doi: 10.1002/smtd.70970 (PMC13599699; doi:10.1002/smtd.70970)
Supplement: Supplementary file 1 [file SMTD-10-e70970-s001.pdf]

SUPPORTING INFORMATION

Impact of plasma ashing on mixed monolayer doping of silicon

Pol Torres-Vila,[*,†] Thilo Glatzel,[‡] Mounir Mensi,[§] Giovanni Boero,[†] Juergen Brugger,[†] Arnaud Bertsch[†]*

[†]Microsystems Laboratory, École Polytechnique Fédérale de Lausanne (EPFL), 1015 Lausanne, Switzerland

[‡] Department of Physics, University of Basel, Klingelbergstrasse 82, 4056 Basel, Switzerland

[§]X-Ray Diffraction and Surface Analytics Platform (XRD-SAP), EPFL Valais, ISIC, Rue de l'Industrie 17, 1951 Sion, Switzerland

*E-mail: pol.torresvila@epfl.ch

S1. Work function calculation and doping model

The work function of silicon was calculated assuming flat-band conditions, following standard semiconductor theory[1]. For p-type silicon, the work function is given by:

$$\Phi_{Si,p} = \chi + \frac{E_g}{2} + k_B T \ln\left(\frac{N_A}{n_i}\right) \quad (1)$$

where χ is the electron affinity of silicon, E_g is the bandgap energy, k_B is the Boltzmann constant, T is the absolute temperature, N_A is the acceptor concentration, and n_i is the intrinsic carrier concentration[1].

For n-type silicon, the work function is expressed as:

$$\Phi_{Si,n} = \chi + \frac{E_g}{2} - k_B T \ln\left(\frac{N_D}{n_i}\right) \quad (2)$$

To account for the coexistence of acceptors and donors, a compensation model was used. The effective doping concentration is defined as[1]:

$$N_{eff} = |N_D - N_A| \quad (3)$$

If $N_D > N_A$, the material is n-type and the corresponding expression is used. If $N_D < N_A$, the material remains p-type.

The surface dopant dose was assumed to scale linearly with the molar fraction of ADP in the precursor solution. Therefore, the total dopant dose Q (in atoms/cm^2) can be expressed as:

$$Q = f_{ADP} \cdot Q_{max} \quad (4)$$

where f_{ADP} is the molar fraction of ADP (ranging from 0 to 1), and Q_{max} is the maximum surface dose corresponding to 100% ADP (in atoms·cm^{-2}). This approximation assumes a proportional relationship between the precursor composition and the resulting surface coverage.

This assumption is justified as a first-order approximation, although deviations from linearity may arise, as seen on the XPS P/Si ratio results due to differences in adsorption kinetics and steric effects.

The donor concentration N_D was estimated assuming diffusion from a limited surface source following Fick's second law[2,3]:

$$C(x,t) = \frac{Q}{\sqrt{\pi D t}} \exp\left(-\frac{x^2}{4Dt}\right) \quad (5)$$

where $C(x,t)$ is the dopant concentration at depth x (in cm) and time t (in s), Q is the total dopant dose per unit area (in atoms·cm^{-2}), D is the diffusion coefficient (in cm^2·s^{-1}), and t is the diffusion time

From this expression, the characteristic diffusion length can be estimated as[2,3]:

$$L = \sqrt{Dt} \quad (6)$$

where L is the characteristic diffusion length of the dopant profile.

For the present processing conditions, this corresponds to a diffusion depth on the order of a few nanometers to a few tens of nanometers, depending on the assumed diffusivity.

Since KPFM probes only the near-surface region, the effective donor concentration was calculated by averaging as:

$$N_D^{eff} = \frac{1}{x_{probe}} \int_0^{x_{probe}} C(x)\, dx \quad (7)$$

where N_D^{eff} is the effective donor concentration probed by KPFM, obtained by averaging the dopant concentration profile C(x) (atoms·cm^{-3}) over the probing depth, x_{probe}.

KPFM probes only the near-surface region (typically within a few nanometers), due to its strong surface sensitivity[4]. Therefore, a probing depth of x_{probe} = 2 nm was assumed

S2. Impact of non-uniform carrier distribution on Hall sheet density extraction

In the main text, the sheet carrier density is extracted assuming a uniform carrier distribution across the conductive layer. However, due to the surface-limited nature of the doping process, the actual carrier profile is expected to vary along the depth[5].

To assess the validity of this approximation, a depth-dependent transport model was implemented. The carrier concentration profile was described using the Fick diffusion solution for a limited surface source[2]:

$$n(z) = \frac{\eta N_{surf}}{\sqrt{\pi D t}} \exp\left(-\frac{z^2}{4Dt}\right) \tag{8}$$

where N_{surf} is the initial surface dose, η is the electrical activation efficiency, D is the diffusivity, and t is the diffusion time.

The carrier mobility was assumed to depend on the local carrier concentration according to a Caughey–Thomas relation[6]:

$$\mu(n) = \mu_{min} + \frac{\mu_{max} - \mu_{min}}{1 + \left(\frac{n}{N_{ref}}\right)^{\alpha}} \tag{9}$$

where μ_{min} and μ_{max} are the minimum and maximum carrier mobilities, respectively, N_{ref} is a reference carrier concentration, and α is an empirical fitting parameter describing the doping dependence of the mobility.

For a system with depth-dependent carrier concentration and mobility, the Hall coefficient can be expressed as[7]:

$$R_H = \frac{\int_0^t n(z)\mu(z)^2 dz}{q\left(\int_0^t n(z)\mu(z) dz\right)^2} \tag{10}$$

where q is the elementary charge and the integration is performed over the thickness t of the conductive silicon region. This expression accounts for the mobility-weighted contribution of carriers along the depth.

The corresponding sheet carrier density is then obtained from:

$$n_S = \frac{1}{qR_H} \tag{11}$$

which leads to:

$$n_s = \frac{\left(\int_0^t n(z)\mu(z) dz\right)^2}{q\int_0^t n(z)\mu(z)^2 dz} \tag{12}$$

The integrals were evaluated numerically, and the resulting sheet carrier density was compared with the value obtained under the uniform carrier distribution approximation. In this case, the sheet carrier density is extracted directly from the Hall voltage slope as:

$$n_{s,\mathrm{uniform}} = \frac{I}{q\left|\frac{dV_H}{dB}\right|} \tag{13}$$

where I is the applied current, q is the elementary charge, and $\frac{dV_H}{dB}$ is the slope of the Hall voltage as a function of the magnetic field.

The comparison shows that the difference between both approaches remains below ~10% for the present experimental conditions, indicating that the uniform approximation provides a reliable estimate of the sheet carrier density. This formulation assumes a single dominant carrier type.

In the non-uniform model, the carrier mobility was described using a Caughey–Thomas relation (Equation (9)) with parameters μ_{max} = 1414 $cm^2 \cdot V^{-1} \cdot s^{-1}$, μ_{min} = 68.5 $cm^2 \cdot V^{-1} \cdot s^{-1}$, $N_{ref} = 9.2 \times 10^{16}$ cm^{-3}, and α = 0.711, as reported in the literature[5] for bulk silicon at room temperature.

S3. Estimation of phosphorus surface density from XPS

In the main text, the phosphorus surface density extracted from the XPS measurements is compared with the sheet carrier density extracted from Hall measurements.

To extract these values from the XPS measurements, a first approximation consists of converting the measured P/Si atomic ratio into a surface density (in atoms·cm^{-2}) using the atomic density of Si (100):

$$N_P = \left(\frac{P}{Si}\right) N_{Si} \tag{14}$$

where $N_{Si} \approx 6.78 \times 10^{14}$ atoms/cm^2.

However, the Si 2p signal includes contributions from subsurface layers due to electron attenuation effects. The measured intensity follows:

$$I(z) = I_0 \exp\left(-\frac{z}{\lambda \cos \theta}\right) \tag{15}$$

where $I(z)$ is the photoelectron intensity originating from a depth z, I_0 is the initial intensity at the surface (i.e., at z=0), λ is the inelastic mean free path of the photoelectrons in the material, and θ is the photoelectron emission angle with respect to the surface normal.

As a result, the silicon signal is overestimated relative to the phosphorus signal.

To account for this effect, a correction factor was introduced:

$$N_P^{corr} = f\left(\frac{P}{Si}\right) N_{Si} \tag{16}$$

The correction factor f accounts for the fact that the Si 2p signal detected by XPS originates not only from the topmost surface layer but from a finite depth, causing the silicon signal to be overestimated relative to the phosphorus signal, which is confined to the surface monolayer.

As described by the attenuation law in Equation (15), the XPS signal arises from a finite depth below the surface due to exponential attenuation of photoelectrons. Approximately 95% of the detected intensity originates from the first $\sim 3\lambda cos\theta$ below the surface.

For the Kratos Axis Supra Plus instrument used in this work, measurements were performed with a monochromated Al Kα source ($h\nu$ = 1486.6 eV) in normal emission geometry (θ = 0°). At this geometry, the inelastic mean free path of Si 2p photoelectrons (kinetic energy ~ 1-1.4 keV) in silicon is, as reported in literature[8,9], on the order of 2-3.5 nm. The effective XPS sampling depth is therefore ~3λ ≈ 6 – 10 nm.

Assuming that the ADP/1-undecene monolayer has a thickness of around 1 nm. The correction factor can be approximated as:

$$f = \frac{3\lambda cos\theta}{d} = \frac{3 \cdot (2 - 3.5\, nm)}{1\, nm} \approx \frac{6 - 10\, nm}{1\, nm} \quad (17)$$

Therefore, the correction factor *f* should not be treated as an exact correction but as an order-of-magnitude estimate and is expected to lie on the range of approximately 6 to 10.

Applying such a correction would bring the XPS- and Hall-derived sheet carrier density values (in atoms·cm^{-2}) closer to each other.

S4. Estimation of the phosphorus diffusion profile under full ADP coverage

To estimate the phosphorus diffusion profile, the limited-source diffusion model introduced in Equation (5) was evaluated assuming full ADP monolayer coverage (100% ADP). Under this assumption, the maximum surface dose was estimated assuming a molecular footprint of ADP (1.1 x 0.8 nm^2) as reported in literature[10].

Using the annealing conditions employed in this work (1050 °C, 10 s), the calculated phosphorus profile predicts a maximum near-surface concentration on the order of 10^{20} cm^{-3}, followed by a rapid decay over the first few tens of nanometers.

If the diffusion depth is defined as the depth at which the phosphorus concentration falls below 10^{10} cm^{-3}, using this criterion, the calculated phosphorus profile extends to approximately 50 nm.

Figure S1 shows the calculated phosphorus diffusion profile obtained from Equation (5) assuming full ADP monolayer coverage.

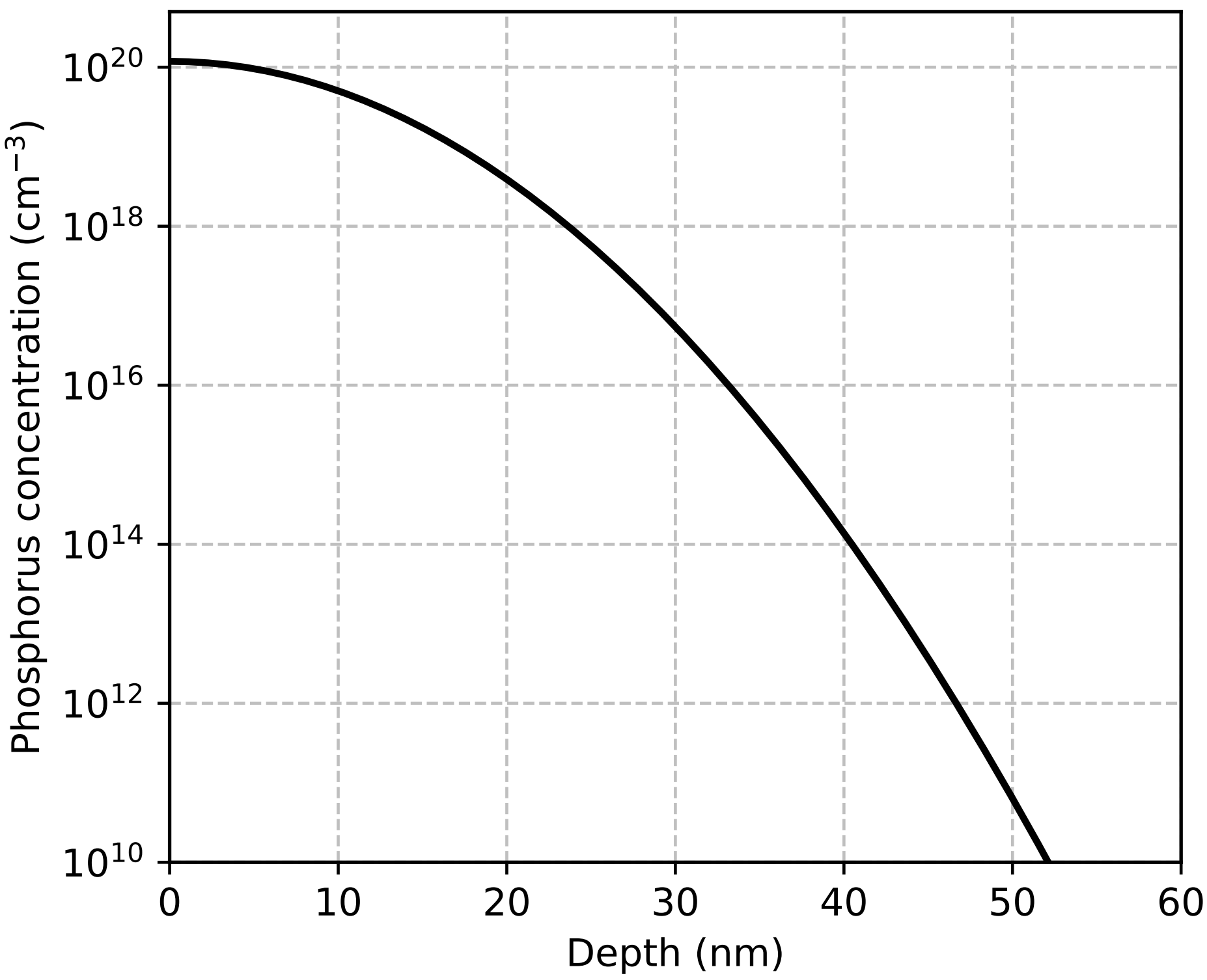

Figure S1. Calculated phosphorus diffusion profile in silicon obtained from the limited-source diffusion model of Eq. (5), assuming full ADP monolayer coverage (100% ADP). The profile was computed for an annealing temperature of 1050 °C and a diffusion time of 10 s.

However, to further analyze the spatial distribution of the dopant dose, the normalized integrated dopant dose was calculated from the diffusion profile shown in Figure S1. This quantity corresponds to the cumulative fraction of the total dopant dose contained within a given depth, obtained by integrating the concentration profile and normalizing it to the total dose:

$$F(x) = \frac{\int_0^x C(x')\,dx'}{\int_0^\infty C(x')\,dx'} \tag{18}$$

where $C(x')$ is the dopant concentration as a function of depth x, x' is the dummy integration variable and $F(x)$ represents the fraction of the total dopant dose contained within a depth x. For the limited-source diffusion profile described by Equation (5), the denominator corresponds to the total dopant dose Q.

Figure S2 shows that a large fraction of the dopant dose is confined within the near-surface region. In particular, approximately 50% of the total dopant dose is located within the first ~5 nm, while 90% and 95% of the dose are contained within ~12.6 nm and ~15 nm, respectively.

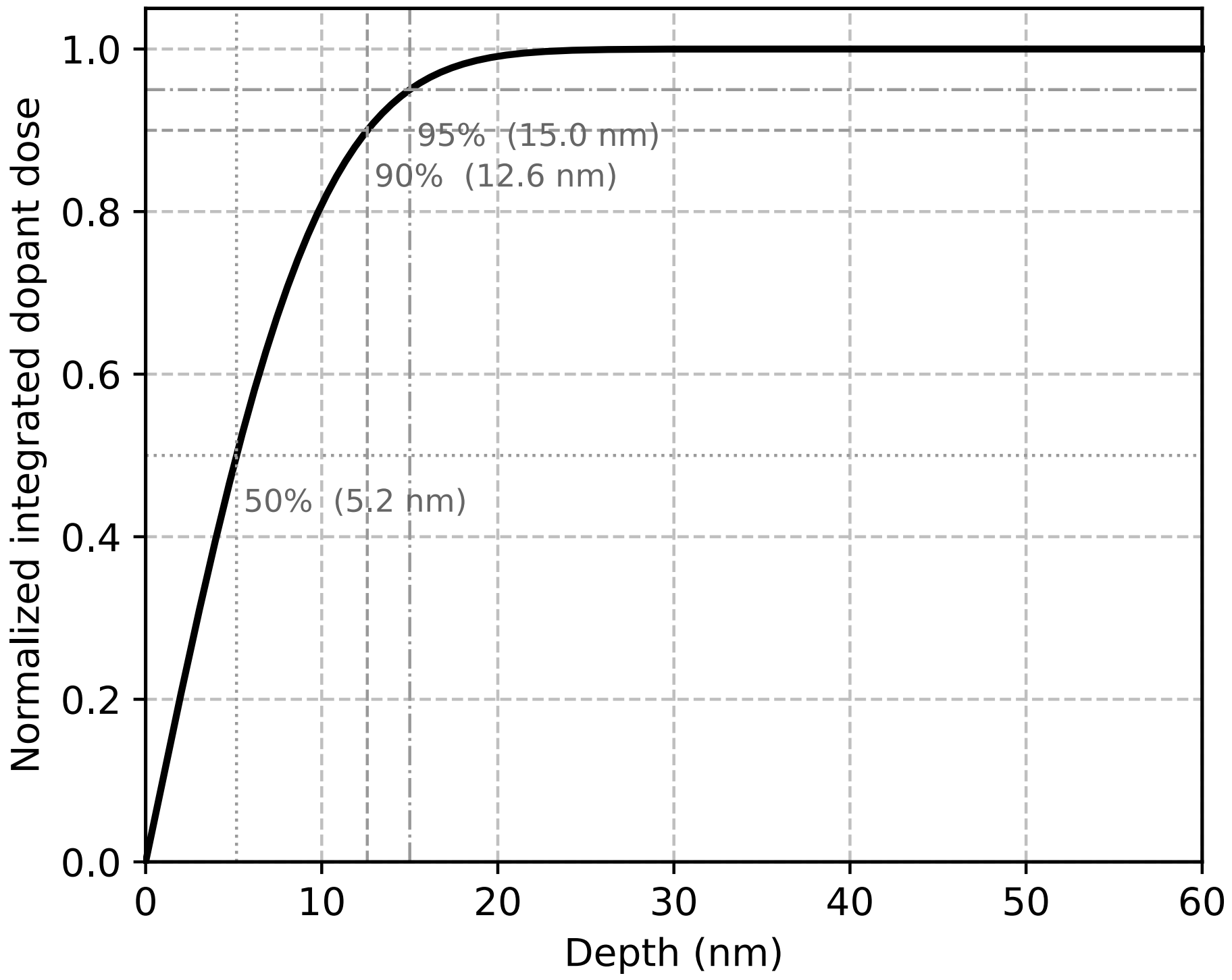

Figure S2. Normalized integrated dopant dose as a function of depth, calculated from the phosphorus diffusion profile shown in Figure S1. The curve represents the cumulative fraction of the total dopant dose contained within a given depth. Characteristic depths corresponding to 50%, 90%, and 95% of the total dose are indicated.

S5. Estimation of carrier mobility and effective dopant concentration from a theoretical diffusion profile

To estimate the carrier mobility associated with the dopant distribution, a theoretical phosphorus diffusion profile was first calculated assuming a full monolayer coverage (100% ADP) at the silicon surface (Figure S1). The diffusion profile was modeled using a limited-source solution of Fick's second law as described in section S4.

The cumulative integrated dopant dose was then computed as a function of depth and normalized to the total dose. From this analysis, the depth containing 95% of the total dopant dose was determined to be approximately 15 nm (Figure S2). This value was used as a characteristic effective thickness of the doped region.

The corresponding sheet dopant density was obtained by integrating the concentration profile from the surface down to this depth:

$$N_{surf} = \int_0^{15\ nm} C(x)dx \tag{19}$$

resolving this integral we get: $N_{surf} = 1.079 \times 10^{14}$ atoms/cm^2

Using this value together with the experimentally measured sheet resistance (R_{sheet}) for a 100% ADP sample from the four-point probe measurements (for example Rsheet (ADP-Grafted – Evaporated SiO_2) = 471.8 ± 0.3 Ω), the carrier mobility can be estimated through:

$$\mu = \frac{1}{qN_{surf}R_{sheet}} \tag{20}$$

where q is the elementary charge.

With this, the electron mobility was extracted obtaining a value of 122.59 cm^2/Vs

Then this mobility was used to determine the equivalent bulk carrier concentration. This was done by using the Caughey-Thomas mobility model (Equation (9)) which describes the dependence of carrier mobility on dopant concentration. This resulted in an effective doping level of:

$N \approx 7.98 \times 10^{18}$ cm^{-3}

See Figure S3 for the simulated diffusion profile and cumulative dopant dose, from which the 95% integration depth (~15 nm) is extracted.

This value lies within the range of the simulated concentration profile in the near-surface region (0–15 nm), although it is slightly lower than the peak concentrations predicted by the model.

This deviation can be attributed to two main factors. First, the present approach assumes that carriers are uniformly distributed within the first 15 nm, corresponding to 95% of the integrated dopant dose. However, the actual dopant distribution is non-uniform, with a concentration gradient from the surface into the bulk. As a result, electrical transport is influenced by regions with lower dopant concentration at larger depths, where carrier mobility is higher. This may lead to an effective mobility that is slightly overestimated within the simplified model.

Second, not all incorporated phosphorus atoms are expected to be electrically active. Incomplete substitutional incorporation, defect-related trapping, or dopant clustering can reduce the fraction of active carriers. As a consequence, the electrically active carrier density is lower than the total dopant dose assumed in the model.

Overall, this approximated analysis provides an order-of-magnitude estimate of both mobility and active dopant concentration.

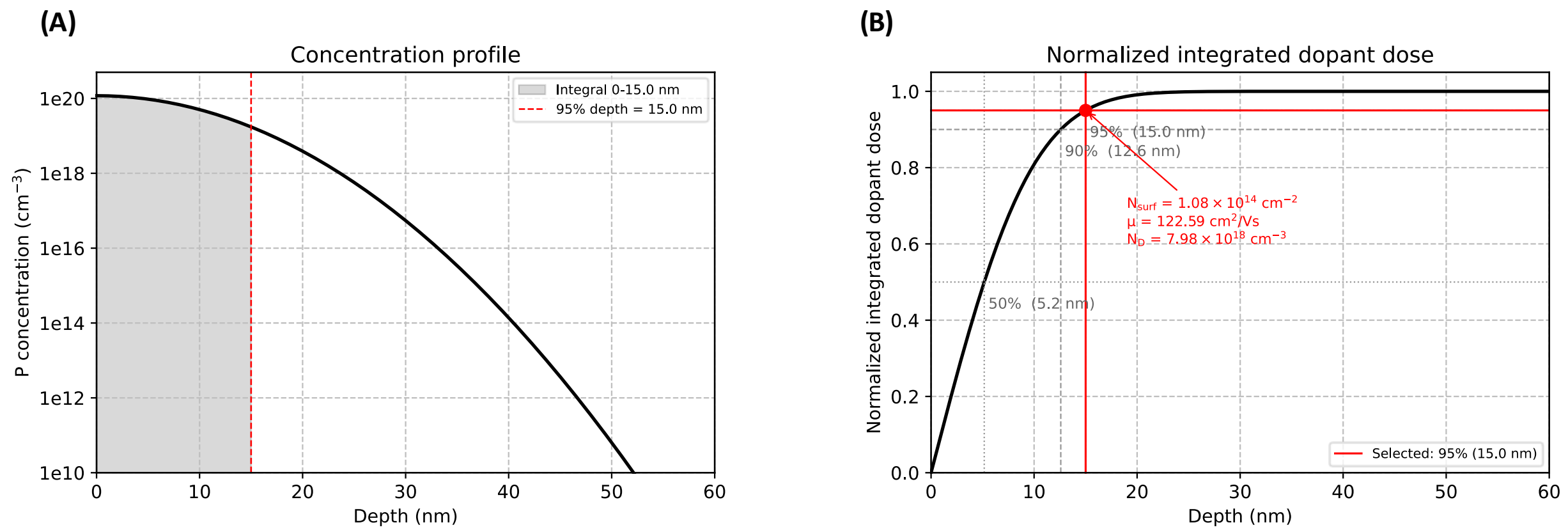

Figure S3. (A) Simulated phosphorus diffusion profile, where the shaded region indicates the integrated dose up to the 95% depth, and (B) corresponding normalized integrated dopant dose used for the extraction of an effective sheet dopant density. The depth containing 95% of the total dopant dose (15 nm) defines an effective doped region, which gives a sheet density of N_{surf} = 1.079×10^{14} cm^{-2}. This value is used to estimate carrier mobility and effective bulk doping from electrical measurements.

S6. Estimation of phosphorus surface density by XPS and Hall effect

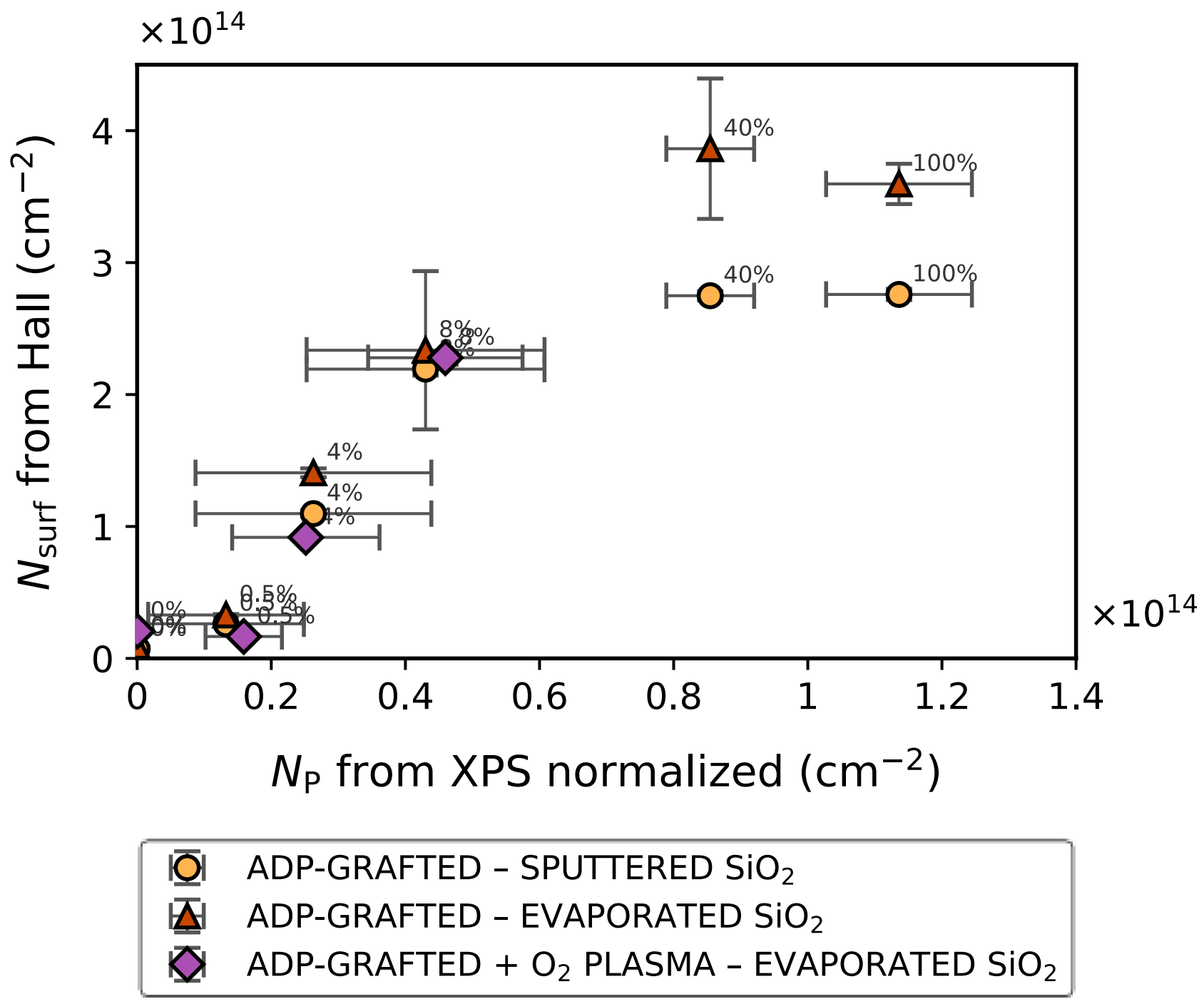

Figure S4. Comparison between the sheet carrier density extracted from Hall measurements (N_{surf}) and the phosphorus surface density estimated from normalized XPS P/Si ratios (N_P). The XPS values are normalized to the 100% ADP condition and scaled using the estimated monolayer coverage (~ 1.1×10^{14} cm^{-2}). Error bars represent the standard deviation (XPS) and measurement uncertainty (Hall).

To further assess the relationship between the sheet carrier density extracted from Hall-effect measurements and the phosphorus surface density prior to diffusion, an additional analysis was performed to estimate the phosphorus surface density from XPS data using a normalized approach.

As discussed in the main text, and section S3, the direct conversion of the measured P/Si ratio into an absolute phosphorus surface density is not straightforward due to attenuation effects and the finite probing depth of XPS, which leads to a systematic underestimation of the true surface concentration. In addition, the P/Si ratio does not scale linearly with the ADP molar fraction due to competitive surface binding between ADP and 1-undecene molecules during monolayer formation.

To partially overcome these limitations, the P/Si ratios obtained for each condition were normalized with respect to the corresponding 100% ADP sample. This normalization assumes that the 100% ADP case approximates a fully covered monolayer, allowing the relative variation of the phosphorus surface density to be extracted independently of absolute XPS sensitivity factors.

Using this approach, the phosphorus surface density for each ADP molar fraction was estimated as:

$$N_P(\%) = \frac{(P/Si)\ (\%)}{(P/Si)\ (\%)(100\%)}\ x\ N_{P,max} \tag{21}$$

where $N_{P.max}$ corresponds to the maximum achievable surface density of phosphorus atoms for a fully covered ADP monolayer. This value was estimated from the molecular footprint of ADP ($1.1 \times 0.8\ nm^2$), yielding a surface density on the order of $1.136 \times 10^{14}\ cm^{-2}$

The resulting XPS-derived phosphorus surface densities were then compared to the sheet carrier densities extracted from Hall-effect measurements. The comparison is shown in Figure S3, where N_{surf} from Hall is plotted as a function of the normalized XPS-derived phosphorus density.

A clear positive correlation is observed between both quantities, with all data points lying within the same order of magnitude (around 10^{13} - $10^{14}\ cm^{-2}$).

However, the XPS-derived values are systematically lower than the Hall-derived carrier densities, by approximately a factor of 3. Factors such as uncertainties in the assumed molecular footprint and full monolayer coverage or possible contributions from additional carriers introduced during processing, as suggested by the non-zero carrier density observed in 0% ADP samples, may contribute to these discrepancies.

Finally, for the 100% ADP case, the extracted phosphorus surface density is consistent with the values obtained from the integrated dopant profiles calculated in Section S5, where the total dose (integrated up to 95% of the dopant distribution) also gives values on the order of $10^{14}\ cm^{-2}$. This further supports the physical consistency of the estimated surface coverage.

S7. P 2s XPS spectra after annealing and capping-layer removal

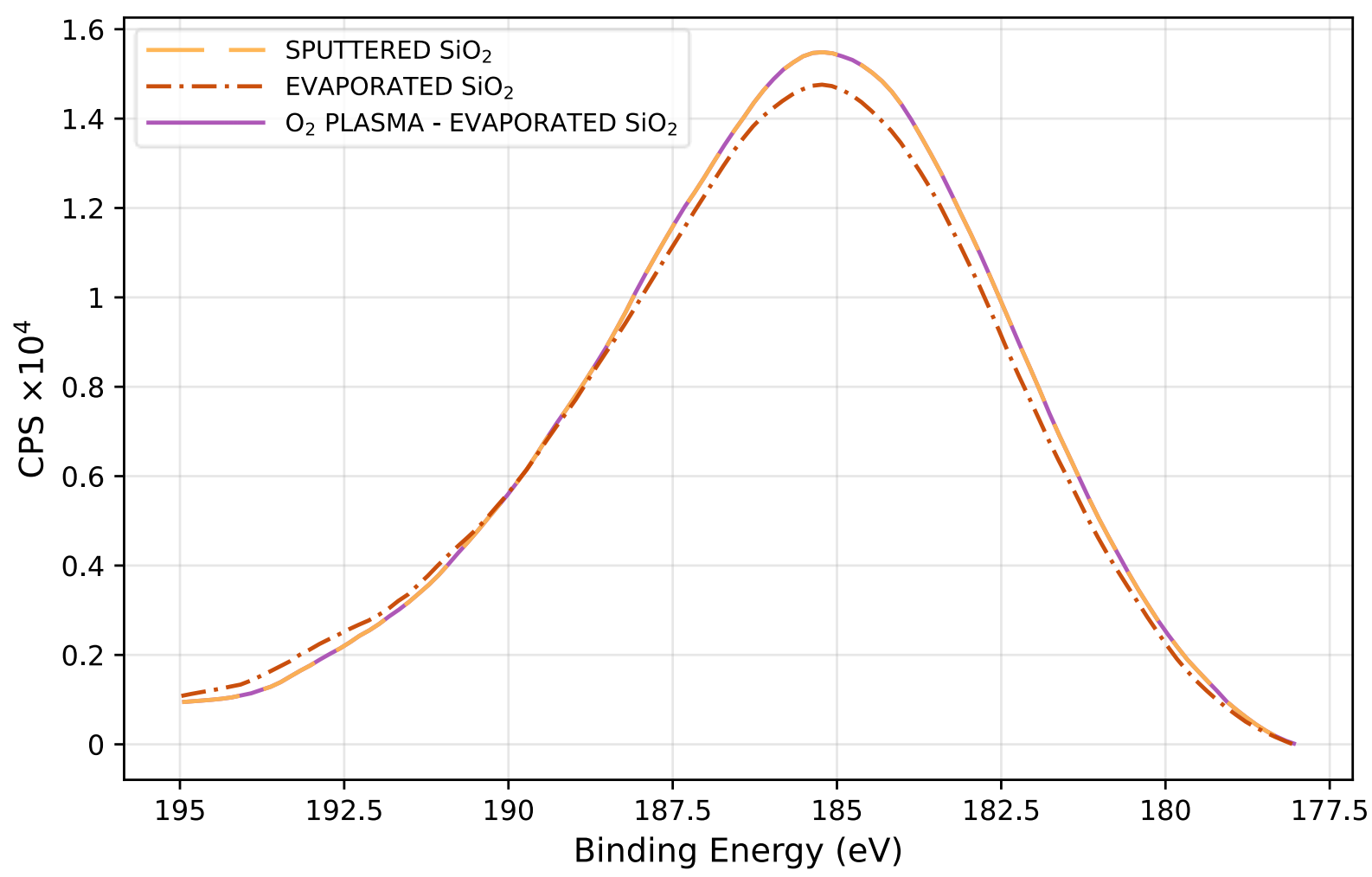

Figure S5. Shirley background subtracted P 2s XPS spectra acquired from samples prepared using 100% ADP (without 1-undecene) after RTA and HF removal of the SiO_2 capping layer. The spectra correspond to samples capped with sputtered SiO_2, evaporated SiO_2, and evaporated SiO_2 after O_2 plasma treatment. No phosphorus peak could be resolved for any of the investigated processing conditions; the measured signal is dominated by the Si plasmon-loss features.

To assess the presence and chemical state of phosphorus after dopant diffusion, XPS measurements were performed on samples prepared using 100% ADP (without 1-undecene) after RTA and HF removal of the SiO_2 capping layer. To increase the acquisition sensitivity, the X-ray source emission current was increased from 15 to 20 mA and the pass energy from 40 to 80 eV. However, as shown in Figure S5 no phosphorus contribution could be resolved in the P 2s region for any of the investigated processing conditions. Depending on the chemical state of phosphorus, a P 2s contribution would be expected within approximately 188-195 eV; however, no peak or shoulder can be distinguished within this range. The measured spectra are dominated by the Si plasmon-loss feature, which overlaps with the expected P 2s signal.

Based on the limited-source diffusion model, the expected near-surface phosphorus concentration after annealing is on the order of 10^{19} cm^{-3}, corresponding to approximately 0.02 at.% relative to the silicon atomic density. This value is below the reported XPS detection limit for phosphorus in silicon under Al Kα excitation, estimated to be approximately 0.3-1 at.%.[11] Together with the overlap between the P 2s signal and the Si plasmon-loss feature, this low phosphorus concentration prevents reliable deconvolution and identification of phosphorus after annealing. Consequently, no reliable assignment of the post-annealing phosphorus chemical state or comparison between the different processing conditions could be performed through an XPS measurement.

SUPPORTING INFORMATION REFERENCES

(1) Sze, S. M.; Ng, K. K. *Physics of Semiconductor Devices*, 3. ed.; Wiley-Interscience online books; Wiley-Interscience: Hoboken, N.J, 2007. https://doi.org/10.1002/0470068329.

(2) Plummer, J. D.; Deal, M. D.; Griffin, P. B. *Integrated Circuit Fabrication: Science and Technology*; Prentice Hall: Upper Saddle River, NJ, 2000.

(3) Franssila, S. *Introduction to Microfabrication*, Repr.; Wiley: Chichester, 2006.

(4) Melitz, W.; Shen, J.; Kummel, A. C.; Lee, S. Kelvin Probe Force Microscopy and Its Application. *Surface Science Reports* **2011**, *66* (1), 1–27. https://doi.org/10.1016/j.surfrep.2010.10.001.

(5) Gao, X.; Guan, B.; Mesli, A.; Chen, K.; Dan, Y. Deep Level Transient Spectroscopic Investigation of Phosphorus-Doped Silicon by Self-Assembled Molecular Monolayers. *Nat Commun* **2018**, *9* (1), 118. https://doi.org/10.1038/s41467-017-02564-3.
(6) Caughey, D. M.; Thomas, R. E. Carrier Mobilities in Silicon Empirically Related to Doping and Field. *Proc. IEEE* **1967**, *55* (12), 2192–2193. https://doi.org/10.1109/PROC.1967.6123.
(7) *The Hall Effect and Its Applications*; Chien, C. L., Westgate, C. R., Eds.; Springer US: Boston, MA, 1980. https://doi.org/10.1007/978-1-4757-1367-1.
(8) Seah, M. P.; Dench, W. A. Quantitative Electron Spectroscopy of Surfaces: A Standard Data Base for Electron Inelastic Mean Free Paths in Solids. *Surface & Interface Analysis* **1979**, *1* (1), 2–11. https://doi.org/10.1002/sia.740010103.
(9) Powell, C. J. Practical Guide for Inelastic Mean Free Paths, Effective Attenuation Lengths, Mean Escape Depths, and Information Depths in x-Ray Photoelectron Spectroscopy. *Journal of Vacuum Science & Technology A: Vacuum, Surfaces, and Films* **2020**, *38* (2), 023209. https://doi.org/10.1116/1.5141079.
(10) Kennedy, M. N. Monolayer Doping of Bulk and Thin Body Group IV Semiconductors. PhD Thesis, University College Cork, Cork, Ireland, 2020. http://hdl.handle.net/10468/10941.
(11) Shard, A. G. Detection Limits in XPS for More than 6000 Binary Systems Using Al and Mg Kα X-rays. *Surface & Interface Analysis* **2014**, *46* (3), 175–185. https://doi.org/10.1002/sia.5406.
